# Cr(vi) removal performance of activated carbon (CB-AC) and nZVI-modified activated carbon (nZVI@CB-AC) derived from waste cigarette butts in aqueous solutions

**DOI:** 10.1039/d6ra03709g

**Published:** 2026-08-04

**Authors:** Türkay Duru, Gözde Duman, Yasemin İşlek Coşkun, Nur Aksuner

**Affiliations:** a Department of Chemistry, Faculty of Science, Ege University 35040 Bornova Izmir Turkey turkay.duru@ege.edu.tr

## Abstract

This study presents and compares two highly efficient adsorbents derived from waste cigarette butts for the removal of Cr(vi) from aqueous solutions using activated carbon (CB-AC) and nZVI-modified activated carbon (nZVI@CB-AC). Both adsorbents achieved 96–99% Cr(vi) removal at a low amount of 0.03 g under optimal conditions at pH 3. The CB-AC and nZVI@CB-AC adsorbents exhibited adsorption capacities of 101.01 mg g^−1^ and 105.26 mg g^−1^, respectively. Various characterization analyses revealed the structural properties of the adsorbents and showed a high surface area. The adsorption data of the nZVI@CB-AC fitted best with the Freundlich model, as indicated by a high correlation coefficient (*R*^2^ = 0.997). Thermodynamic analyses indicated a spontaneous and endothermic process accompanied by increased randomness at the solid–liquid interface. Selectivity studies showed that CB-AC exhibited a high Cr(vi) removal efficiency even in the presence of competing ions. XPS analysis revealed that the predominant removal mechanism was a redox interaction between Cr(vi) ions and nZVI@CB-AC, which contributed to the adsorbent's higher capacity, faster kinetics, and improved efficiency. Overall, this research emphasized both CB-AC and nZVI@CB-AC as promising and effective adsorbents for Cr(vi) removal from aqueous environments.

## Abstract 2

This study presents and compares two highly efficient adsorbents derived from waste cigarette butts for the removal of Cr(vi) from aqueous solutions using activated carbon (CB-AC) and nZVI-modified activated carbon (nZVI@CB-AC).

## Graphical abstract

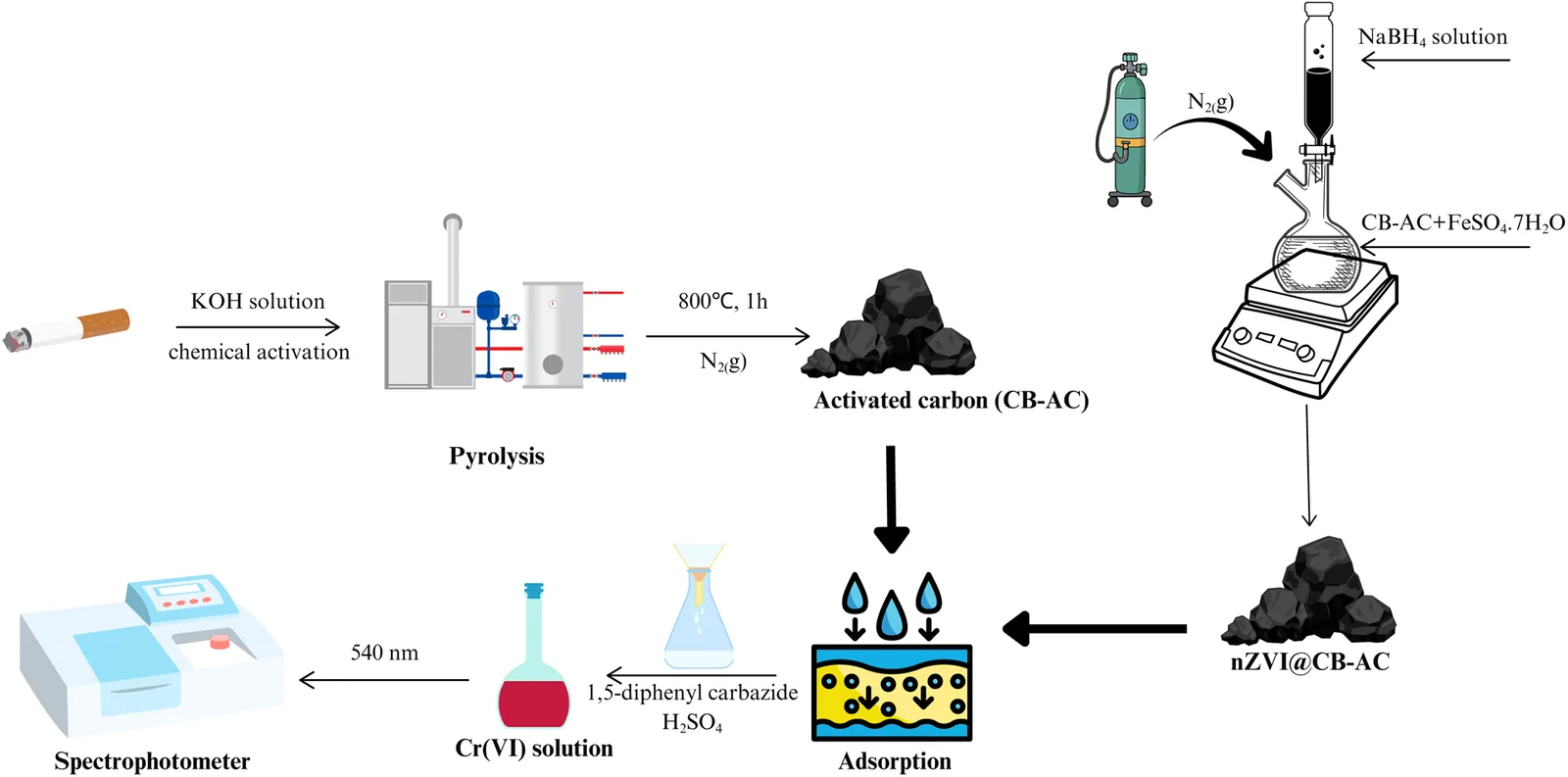

## 1 Introduction

Heavy metal ions are among the most common environmental pollutants and thus represent a significant ecological and public health concern. Heavy metals are not biodegradable; therefore, they tend to bioaccumulate, with their concentrations increasing within living organisms over time. These toxic metallic elements are continuously discharged into aquatic systems from various natural and anthropogenic sources.^1^ Among these toxic metals, chromium (Cr) is a pollutant of primary concern, which is particularly emitted from industrial sources such as electroplating, pigment production, and leather tanning.^2^ Chromium has two oxidation states: trivalent (Cr(iii) and hexavalent (Cr(vi)). The trivalent form is assumed to be an essential micronutrient for humans and some animals, whereas the hexavalent form is highly toxic and carcinogenic. According to the World Health Organization, the concentration of Cr(vi) in drinking water should not exceed 0.05 mg L^−1^.^3^ Therefore, removal of Cr(vi) from water used for drinking purposes is crucial. Various removal techniques, including ion exchange, coagulation/precipitation, membrane separation processes, chemical precipitation, biological treatment methods, electrocoagulation, photocatalytic reduction,^4,5^ adsorption, and biosorption, have been developed for the removal of chromium from wastewater for ages.^6^ Among these methods, adsorption is preferred owing to its high efficiency, flexibility, and simplicity in both design and operation.

Activated carbon (AC) is one of the most widely used adsorbents due to its high specific surface area and surface functional groups. AC provides several advantages, including the ability to be modified for higher selectivity towards the analyte, the enhancement of porosity and surface area through activation processes. The activation process can be carried out by using physical or chemical methods. In this study, chemical activation was performed using potassium hydroxide (KOH) as a chemical activating agent.^7^ KOH provides a significant effect on the surface area and porosity of the resulting activated carbon.^8^ Hence, AC can be effectively used in wastewater treatment processes and for the removal of heavy metals such as chromium from aqueous solutions.

Agricultural by-products (*e.g.*, coconut shells, rice husks, and fruit stalks) and other biomass sources (*e.g.*, wood residues and algae) are commonly used as raw materials for activated carbon production.^7^ Recently, the use of environmental waste and industrial by-products for the preparation of activated carbon has increased.^9,10^ Nowadays, cigarette butts (CB) are the most encountered anthropogenic waste in the environment. Disposal of non-degradable waste, especially CBs, into the environment is a significant global pollution problem. Therefore, converting these wastes into reusable forms is essential in order to prevent environmental pollution.^11,12^

Nanoparticles have also been used as adsorbents due to their high adsorption capacities for a wide variety of pollutants, especially for heavy metals and organic contaminants, which are attributed to their large specific surface areas and high surface reactivity. Iron-modified activated carbons have been identified as highly efficient adsorbents because of their facile synthesis, the wide availability of biomass-based precursors, and their capability to host diverse iron species, including zero-valent iron (Fe°) and magnetite (Fe_3_O_4_).^13^ Nano zero-valent iron (nZVI) possesses strong reducing power, high reactivity, and a large specific surface area, which make it particularly effective for chromium remediation. In chromium-contaminated systems, nZVI enables the simultaneous adsorption and reductive transformation of Cr(vi) into the less toxic and less mobile Cr(iii).^14^ The reduction of Cr(vi) by nZVI primarily is carried out *via* direct electron transfer from Fe^0^ to Cr(vi), resulting in the formation of Fe^2+^ and Cr(iii), as depicted following reactions:^15^

In acidic conditions:

1 2HCrO_4_^−^ + 3Fe^0^ + 14H^+^ → 2Cr^3+^ + 3Fe^2+^ + 8H_2_O

2 HCrO_4_^−^ + 3Fe^2+^ + 7H^+^ → 2Cr^3+^ + 3Fe^3+^ + 4H_2_O

In alkaline conditions:

3 2CrO_4_^−^ + 3Fe^0^ + 16H^+^ → 2Cr^3+^ + 3Fe^2+^ + 8H_2_O

4 2CrO_4_^−^ + 3Fe^2+^ + 8H^+^ → 2Cr^3+^ + 3Fe^2+^ + 4H_2_O

5 *x*Cr^3+^ + (1 − *x*)Fe^3+^ + 3H_2_O → Cr_*x*_Fe_(1−*x*)_(OH)_3_ + 3H^+^

Compared with other metallic reductants, nZVI offers several advantages, including low toxicity, low cost, and ease of production. However, due to its small particle size, it tends to agglomerate and oxidize easily, which limits its effectiveness. To overcome these disadvantages, the immobilization of nZVI particles in a porous matrix has been considered and used to improve their dispersion and thereby enhance their reactivity.^16^

Recently, Qiu *et al.* demonstrated that sludge-derived biochar enriched with nano zero-valent iron (nZVI-SDBC) showed significantly higher efficiency in removing Cr(vi) and Cr(iii) than that of raw biochar.^17^ nZVI-SDBC not only enhanced adsorption capacity but also effectively reduced Cr(vi) to Cr(iii). Dermawan *et al.* reported that sugarcane bagasse biochar composited with nano zero-valent iron (nZVI) exhibited superior Cr(vi) removal compared to raw biochar.^18^ The mechanism of Cr(vi) removal was reported to involve adsorption, reduction, and co-precipitation, achieving capacities of 86.5–112.4 mg g^−1^ for composite biochar and 77.8 mg g^−1^ for raw biochar. Adsorption followed the Freundlich isotherm and pseudo-second-order kinetics (PSO), indicating chemisorption on heterogeneous surfaces. Similarly, Gill *et al.* reported that orange peel biochar modified with nZVI achieved higher Cr(vi) removal (94.1%) than raw biochar (71.7%), owing to its larger surface area, porous structure, and reduction of Cr(vi) to Cr(iii).^19^ The influence of coexisting ions was also examined; phosphate ions slightly inhibited Cr(vi) removal, whereas sulfate ions enhanced it by promoting surface reactivity. A study by Deng *et al.* revealed that the Cr(vi) removal using wheat straw biochar modified with nZVI and chitosan may involve multiple mechanisms, including pore adsorption, electrostatic adsorption, and chemical reduction.^20^

Despite these advances, previous studies have primarily focused on the immobilization of nZVI onto biochars derived from conventional biomass and agricultural wastes. In contrast, the present study investigates the preparation of activated carbon from waste cigarette butts (CB-AC) and its modification with nano zero-valent iron (nZVI@CB-AC) for the removal of Cr(vi) from aqueous media. In addition to comparing the Cr(vi) removal performance of CB-AC and nZVI@CB-AC, both residual total Cr and Cr(vi) concentrations were determined, enabling a comprehensive evaluation of chromium speciation and pH-dependent transformation during the removal process. Furthermore, KOH activation produced a highly developed porous structure and a substantially higher specific surface area than those reported for previously developed biochar-based supports, providing abundant active sites for the dispersion and immobilization of nZVI particles. In this study, physicochemical characterization of both CB-AC and nZVI@CB-AC was conducted to evaluate the influence of nZVI incorporation on the structural and surface properties of the activated carbon. The effects of various parameters, such as initial pH, adsorbent dosage, contact time, and initial Cr(vi) concentration on Cr(vi) removal were examined. Under the optimum conditions, both residual Cr(vi) and total chromium concentrations were determined to evaluate chromium speciation and elucidate the contribution of the adsorption–reduction process to Cr(vi) removal. In addition, adsorption isotherms, thermodynamic parameters, and the possible Cr(vi) removal mechanisms were investigated for both adsorbents. Finally, interference experiments and Cr(vi) removal tests using real wastewater samples were conducted to evaluate the practical applicability and selectivity of the adsorbents.

## 2 Materials and methods

### 2.1 Chemicals

Waste CBs, which were used as precursor material for activated carbon, were collected from the streets. All chemicals were of analytical grade. Deionized water was used throughout the study. Iron(ii) sulfate heptahydrate (FeSO_4_·7H_2_O) and sodium borohydride (NaBH_4_), which were used for modification, and potassium dichromate (K_2_Cr_2_O_7_) (source of Cr(vi)), hydrochloric acid (HCl), sodium hydroxide (NaOH), and potassium hydroxide (KOH) were purchased from Merck. Diluted HCl and NaOH solutions were used to adjust the pH. The Cr(vi) stock solution (1.000 mg L^−1^) was prepared by dissolving an aliquot of K_2_Cr_2_O_7_. The model solutions and standards of Cr(vi) were prepared daily. HCl, H_2_SO_4_, HNO_3_, and NaOH was used for the desorption studies. For interference studies, KCl, K_2_SO_4_, NaHCO_3_, Na_3_PO_4_, NaCl, CaCl_2_, and Ni(NO_3_)_2_·6H_2_O salts were used as sources of anions and cations.

### 2.2 Apparatus

The pH of the solutions was adjusted using a pH meter (Mettler Toledo FiveGo FG-2, Germany). Adsorption studies were conducted with a vibration water bath (Nuve ST-402, Turkey) and an orbital shaker operating at 350 rpm (Biosan OS-10, Latvia). Cr(vi) absorption measurements were performed using a PG Instruments TG 80+ model UV-vis spectrophotometer at 540 nm in double beam model. Total Cr determination was carried out by inductively coupled plasma mass spectrometry (ICP-MS, Agilent 7900). The surface functional groups of the adsorbents were identified using Attenuated Total Reflectance Fourier Transform Infrared (ATR-FTIR) spectroscopy in the range of 4000 to 400 cm^−1^ on a PerkinElmer Spectrum Two spectrometer (PerkinElmer 100, USA). Surface morphology and elemental composition were investigated using a Thermo Fisher Scientific Apreo S LoVac (USA) scanning electron microscope (SEM) coupled with an energy-dispersive X-ray (EDX) spectrometer. Before imaging, the samples were sputter-coated with a thin gold layer to ensure conductivity. The specific surface area, pore volume, and pore size distribution were determined from nitrogen adsorption–desorption isotherms measured at 77 K using a Micromeritics 3 Flex analyzer. Before analysis, all samples were degassed under vacuum at 300 °C for 3 hours. The surface elemental composition and chemical states of key elements (C, O, Fe) were examined by X-ray photoelectron spectroscopy (XPS) using a Thermo Scientific K-Alpha+ spectrometer with a monochromatic Al Kα X-ray source.

### 2.3 Preparation of adsorbent

Initially, the collected waste CBs were separated from their protective paper coverings and fragmented into small pieces. To facilitate the development of a porous structure within the activated carbon, chemical activation was performed using potassium hydroxide (KOH). The CBs fragments were then mixed with a potassium hydroxide (KOH) solution at a 3 : 1 (g g^−1^) ratio for chemical activation, followed by oven drying. The dried mixture was pyrolyzed at 800 °C for 1 hour under a nitrogen (N_2_) atmosphere. After pyrolysis, the resulting material was neutralized using a 10% hydrochloric acid (HCl) solution in a reflux system. After neutralization, vacuum filtration was performed, and the product was washed with deionized water until the washing solution reached a neutral pH, indicating the removal of residual chlorine. Finally, the material was dried in an oven at 105 °C to obtain the AC.^21^ The obtained material was denoted as CB-AC (cigarette butts activated carbon).

### 2.4 Preparation of nZVI-modified cigarette butts activated carbon (nZVI@CB-AC)

The synthesis of nZVI-modified cigarette butts activated carbon (nZVI@CB-AC) was carried out by chemically reducing zero-valent iron nanoparticles (nZVI) onto the surface of the CB-AC. For this purpose, 1.38 g of FeSO_4_·7H_2_O was dissolved in a 100 mL ethanol/water mixture (30 mL ethanol/70 mL deionized water) to prepare a 0.25 M solution. Subsequently, 1.25 g of CB-AC was added to the FeSO_4_·7H_2_O solution in a two-neck flask under a nitrogen (N_2_) atmosphere, and the mixture was stirred magnetically for 15 minutes. In a separate container, 0.47 g of NaBH_4_ was dissolved in 50 mL of deionized water to prepare a 0.25 M solution, which was then added dropwise to the iron solution at a rate of 2 mL min^−1^ using a dropping funnel. After the complete addition of NaBH_4_, the mixture was stirred for an additional 15 minutes. The resulting material was filtered under vacuum and washed with 200 mL of ethanol to remove residual water. Finally, the product was dried in an oven at 80 °C for 5 hours to obtain the nZVI@CB-AC composite.^22^

### 2.5 Adsorption experiments

For the Cr(vi) adsorption experiments, a 1000 mg L^−1^ Cr(vi) stock solution was initially prepared from K_2_Cr_2_O_7_ salt and working solutions with a concentration of 50 mg L^−1^ were subsequently prepared from this stock. The optimization studies were conducted using 25 mL of solution with an initial Cr(vi) concentration of 50 mg L^−1^, 25 mg of adsorbent, and a contact time of 24 h. After optimization, the adsorbent dosage was determined to be 1 g L^−1^. During the optimization process, the effects of pH, adsorbent dosage, contact time, and temperature were investigated.

Isotherm studies were conducted with initial concentrations ranging from 25 to 250 mg L^−1^, and kinetic models were examined between contact times of 1 and 1440 min. The adsorbent was separated from the sample solutions using filter paper, and the residual Cr(vi) concentrations in the supernatant were measured at 540 nm using a UV-vis spectrophotometer using the 1,5-diphenylcarbazide method.^23^ Briefly, the sample was first acidified with sulfuric acid (H_2_SO_4_) and then reacted with 1,5-diphenylcarbazide to form a purple-colored complex. The absorbance of this complex was measured at 540 nm using a UV-vis spectrophotometer.

The total Cr (Cr(T)) and Fe concentrations were determined ICP-MS. The concentration of Cr(iii) in solution was calculated from the difference between the Cr(T) and Cr(vi) concentrations. All experiments were performed in triplicate, and the mean values of the obtained results were recorded.

The equilibrium adsorption capacity of the adsorbents for Cr(vi) (*q*_e_, mg g^−1^) was calculated using eqn (6), while the Cr(vi) removal efficiency (*R*, %) was determined according to eqn (7):6
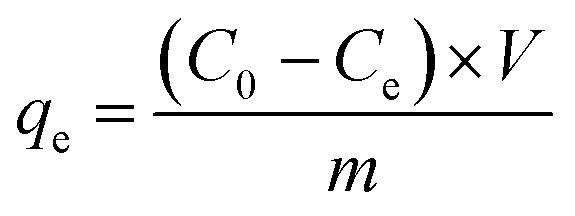
7
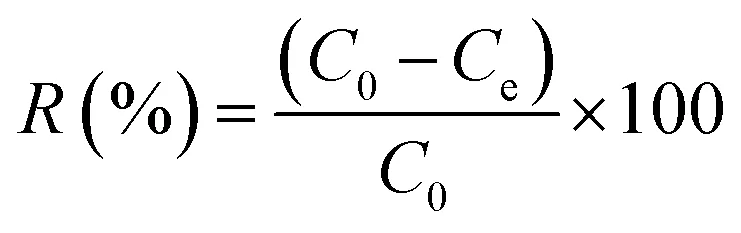
where *C*_0_ and *C*_e_ (mg L^−1^) are the initial and equilibrium Cr(vi) concentrations, respectively; *V* (L) is the solution volume, and *m* (g) is the mass of the adsorbent used.

Langmuir and Freundlich isotherm models were evaluated using nonlinear regression based on their non-transformed equation. The parameter values obtained from the corresponding linearized model were used as initial estimates, and the nonlinear optimization was performed using the Solver tool in Microsoft Excel (GRG Nonlinear algorithm). The model parameters were optimized by minimizing the sum of squared errors (SSE) between the experimental and predicted adsorption capacities. In addition, the root mean square error (RMSE) and SSE values were calculated. Model performance was comparatively assessed using *R*^2^, RMSE, and SSE, with a higher *R*^2^, lower RMSE and SSE values indicating a better fit to the experimental adsorption data.

## 3 Result and discussion

### 3.1 Characterization of CB-AC and nZVI@CB-AC

**Fig. 1:**
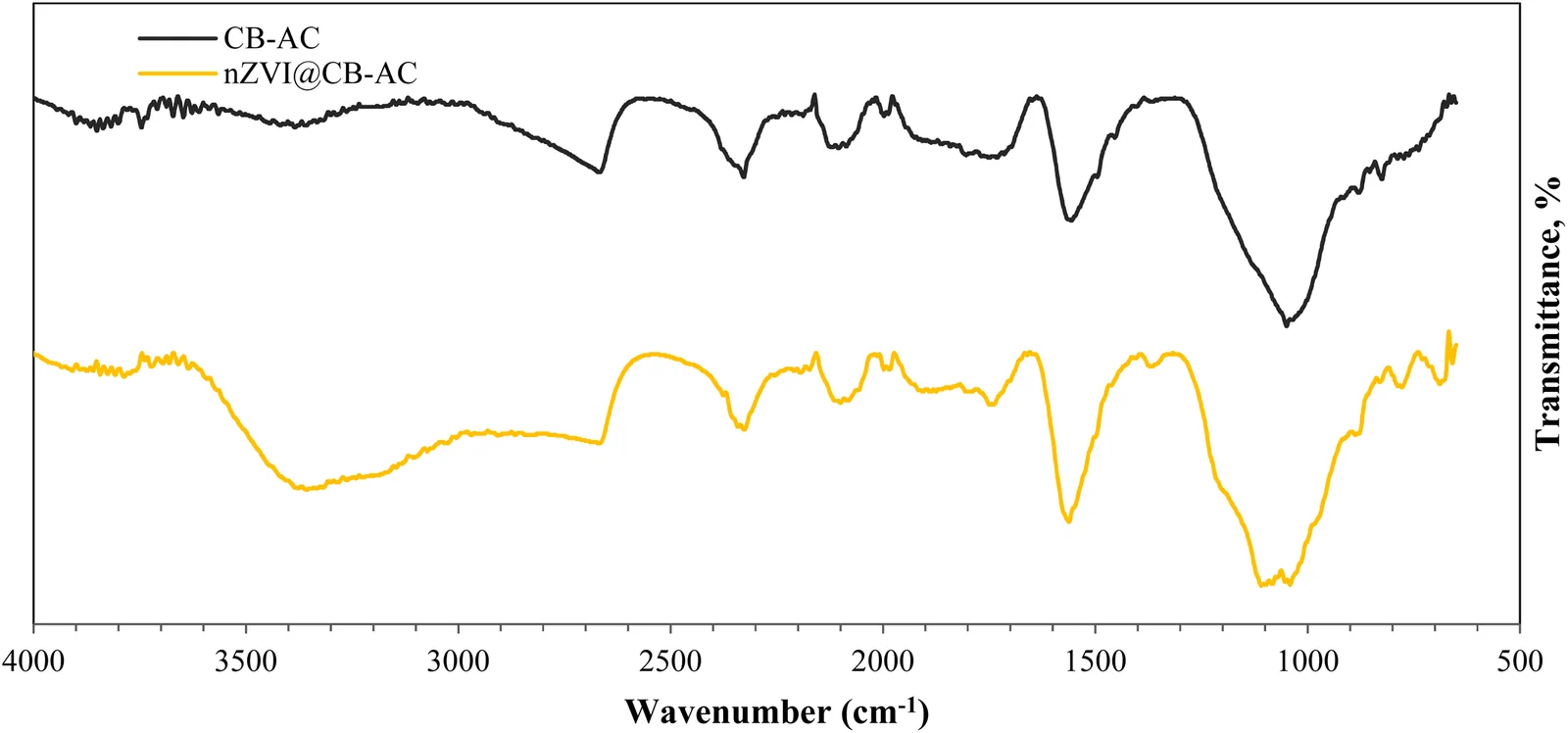
FT-IR spectrum of CB-AC and nZVI@CB-AC.

The surface functional groups of synthesized CB-AC and nZVI@CB-AC, and the interaction between the carbon matrix, belonging to CB-AC and nZVI, were investigated by FTIR spectroscopy, and the obtained spectra are presented in Fig. 1.

In the spectrum of CB-AC, the broad band observed at 3388 cm^−1^ is attributed to the stretching vibrations of hydroxyl (–OH) groups associated with surface phenols, alcohols, and adsorbed moisture. The weak bands between 2700 and 2300 cm^−1^ correspond to aliphatic C–H stretching vibrations, indicating a low content of aliphatic structures and a predominantly aromatic carbon framework. The absorption band at 1556 cm^−1^ is assigned to aromatic C=C stretching and/or conjugated C=O vibrations, which are characteristic of carbonized materials. Additionally, the band observed around 1100–1000 cm^−1^ is attributed to C–O stretching vibrations of alcohol, ether, or phenolic groups present on the CB-AC surface.^19,24^

After modification with nZVI, considerable changes are observed in the spectrum of nZVI@CB-AC. These changes indicated successful modification of nano zero valent iron on the surface of CB-AC. The –OH stretching band at 3361 cm^−1^ becomes more evident, being attributed to hydroxyl groups interacting with iron oxides/hydroxides (Fe–OH) formed during or after nZVI modification. This enhancement suggests increased surface hydroxylation and improved hydrophilicity of the composite material.^25^

Furthermore, the shift of the characteristic bands belonging to C=O and/or C=C at 1600 cm^−1^ to 1563 cm^−1^ indicates a change in the electronic environment around these bands. This phenomenon indicated alterations in the electronic environment of oxygen-containing functional groups arising from chemical interactions (coordination or complexation) between iron species and carboxyl or phenolic groups on the CB-AC surface rather than physical mixing.^26^

After nZVI loading, the band at 1050 cm^−1^, indicating C–O stretching vibrations of phenolic and ether groups, was split into two distinct peaks at 1087 and 1046 cm^−1^. This band splitting could indicate the presence of C–O groups in different chemical environments, which can be attributed to the coordination of iron species with oxygen-containing functional groups on the CB-AC surface. While the band at 1046 cm^−1^ can be associated with Fe–O–C bonds, the band at 1087 cm^−1^ corresponds to non-coordinated C–O groups, confirming interactions between nZVI and the carbon structure of CB-AC.^27,28^ In addition, the appearance of new bands at around 879 and 698 cm^−1^, which are absent in pristine CB-AC, is associated with Fe–O, Fe–O–Fe and Fe–OOH vibrations, confirming the presence of iron-based phases on the AC surface.^25,29^ The appearance of this band provides compelling evidence for the successful loading of iron nanoparticles onto the activated carbon support without drastically altering its fundamental structure. A comparative FTIR analysis reveals distinct changes upon nZVI modification, confirming the successful formation of the composite.

**Fig. 2:**
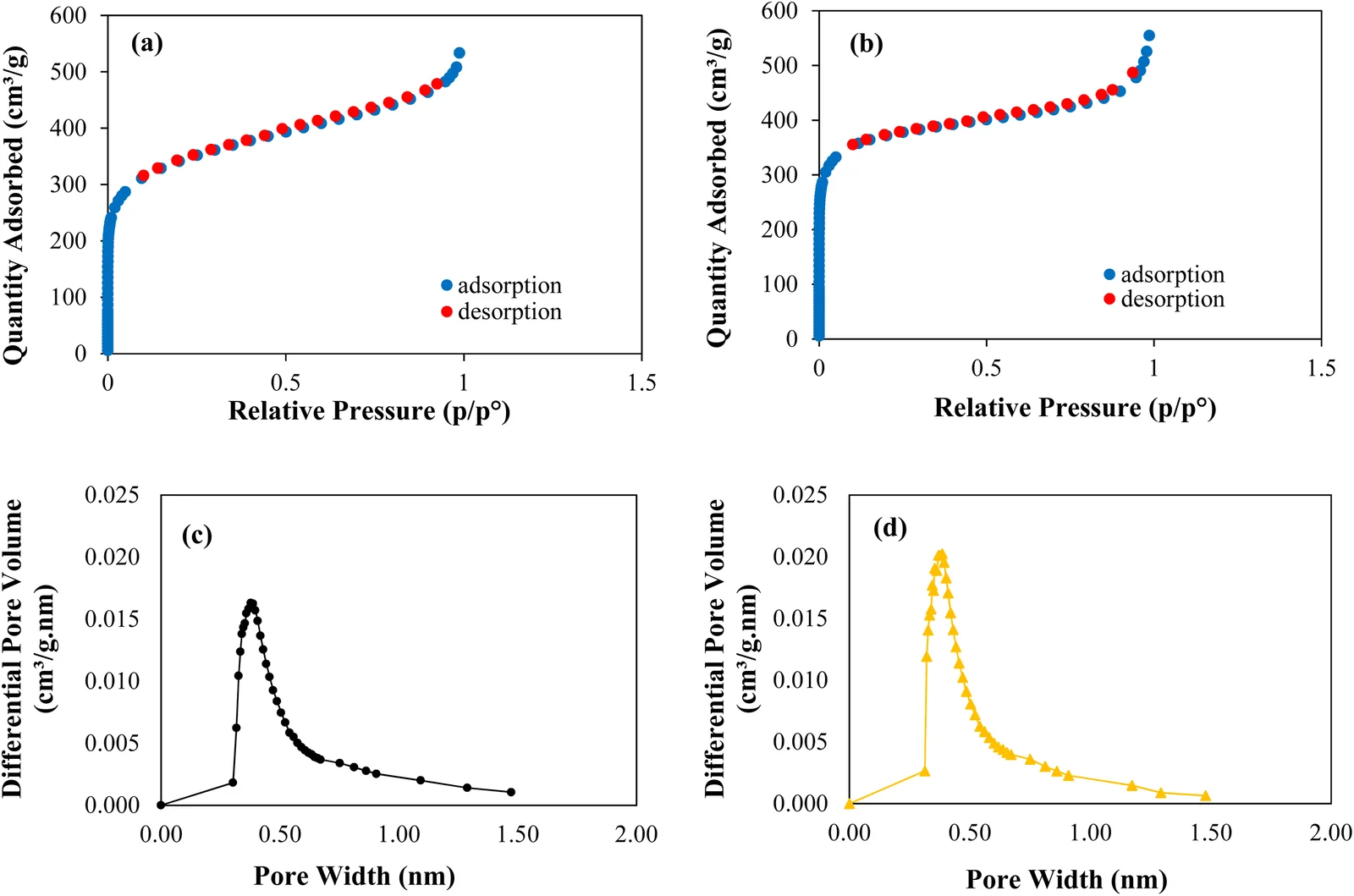
N_2_ adsorption–desorption isotherms of (a) CB-AC and (b) nZVI@CB-AC, and the corresponding Horvath–Kawazoe pore size distribution curves of (c) CB-AC and (d) nZVI@CB-AC.

The Brunauer, Emmett, and Teller (BET) analysis elucidated the surface area, total pore area, total pore volume, and mean pore diameter of the CB-AC. The results are presented in Table 1. The pristine CB-AC exhibited a high BET surface area of 1225.8 m^2^ g^−1^, total pore area of 950.0 m^2^ g^−1^, total pore volume of 0.418 cm^3^ g^−1^, and mean pore diameter of 0.468 nm. The pores with diameters below 2 nm are classified as micropores.^30^ After nZVI modification, the composite demonstrated substantial improvements in its textural properties. The BET surface area of nZVI@CB-AC increased to 1413.7 m^2^ g^−1^, accompanied by a corresponding rise in the total pore area (1246.5 m^2^ g^−1^), total pore volume (0.506 cm^3^ g^−1^), and a decrease in mean pore diameter of 0.434 nm. The Horvath–Kawazoe pore size distribution curves (Fig. 2c and d) confirmed that both samples were predominantly microporous. Although metal nanoparticle deposition is often associated with partial pore blockage, the present results suggest that the microporous structure was largely preserved after nZVI modification. The increased micropore surface area and micropore volume, together with the slight decrease in median pore width, indicate a greater contribution of narrower micropores to the overall pore structure rather than pore blockage. Consequently, the increase in BET surface area is consistent with the presence of narrower micropores, which provide a higher surface area per unit volume. Similarly, Xiong *et al.* reported that nZVI loading does not necessarily cause significant pore blockage and that the porous structure of the support can be largely preserved following metal incorporation.^31^

**Table 1:** The textural parameters of porous carbon adsorbents

Adsorbent	Surface area (m^2^ g^−1^)	Pore area (m^2^ g^−1^)	Pore volume (cm^3^ g^−1^)	Median pore width (nm)
CB-AC	1225.8	950.0	0.418	0.468
nZVI@CB-AC	1413.7	1246.5	0.506	0.434

The textural properties of the synthesized CB-AC and nZVI@CB-AC composites were evaluated using N_2_ adsorption–desorption analysis, with the resulting isotherms presented in Fig. 2. According to the IUPAC classification system, the N_2_ adsorption–desorption isotherms of both adsorbents exhibit Type I characteristics. The sharp uptake at very low relative pressures (*P*/*P*° < 0.15) confirms micropore filling as the dominant adsorption mechanism.^30,32^ This is a definitive signature of a predominantly microporous structure, a characteristic feature of chemically activated carbons, particularly those derived from KOH activation.^33^ In addition, a weak hysteresis loop can be observed at intermediate relative pressures. Such hysteresis is commonly classified as H4-type and is often associated with narrow slit-shaped pores in activated carbons. Notably, H4-type hysteresis frequently accompanies Type I isotherms and is indicative of microporous structures with slit-like pores rather than well-developed mesoporosity.^30^ At higher relative pressures (*P*/*P*^0^ > 0.9), a gradual increase in nitrogen uptake is observed for both samples, suggesting a minor contribution from capillary condensation in larger pores or interparticle voids. Compared with the pronounced hysteresis loops reported for micro–mesoporous carbons in the literature,^8^ the relatively weak hysteresis observed in this study indicates that the pore structure of both CB-AC and nZVI@CB-AC is dominated by micropores, with only a limited mesoporous contribution.

The development of this high-surface area microporous structure during KOH activation can be attributed to a series of redox and etching reactions, as described below:^33^

8 2KOH → K_2_O + H_2_O

9 4KOH + C → 2K_2_O + 2H_2_ + K_2_CO_3_

10 H_2_O + C → CO + H_2_

11 K_2_CO_3_ → K_2_O + CO_2_

12 K_2_CO_3_ + 2C → 2K + 3CO

13 K_2_O + C → 2K + CO

In conclusion, the high specific surface area and well-developed microporosity of both adsorbents, further enhanced by nZVI modification, provide abundant adsorption sites for pollutant adsorption. For the nZVI@CB-AC composite, this superior porous structure not only facilitates adsorption but also ensures the dispersion and accessibility of the iron active sites, synergistically contributing to its high Cr(vi) removal potential.

**Fig. 3:**
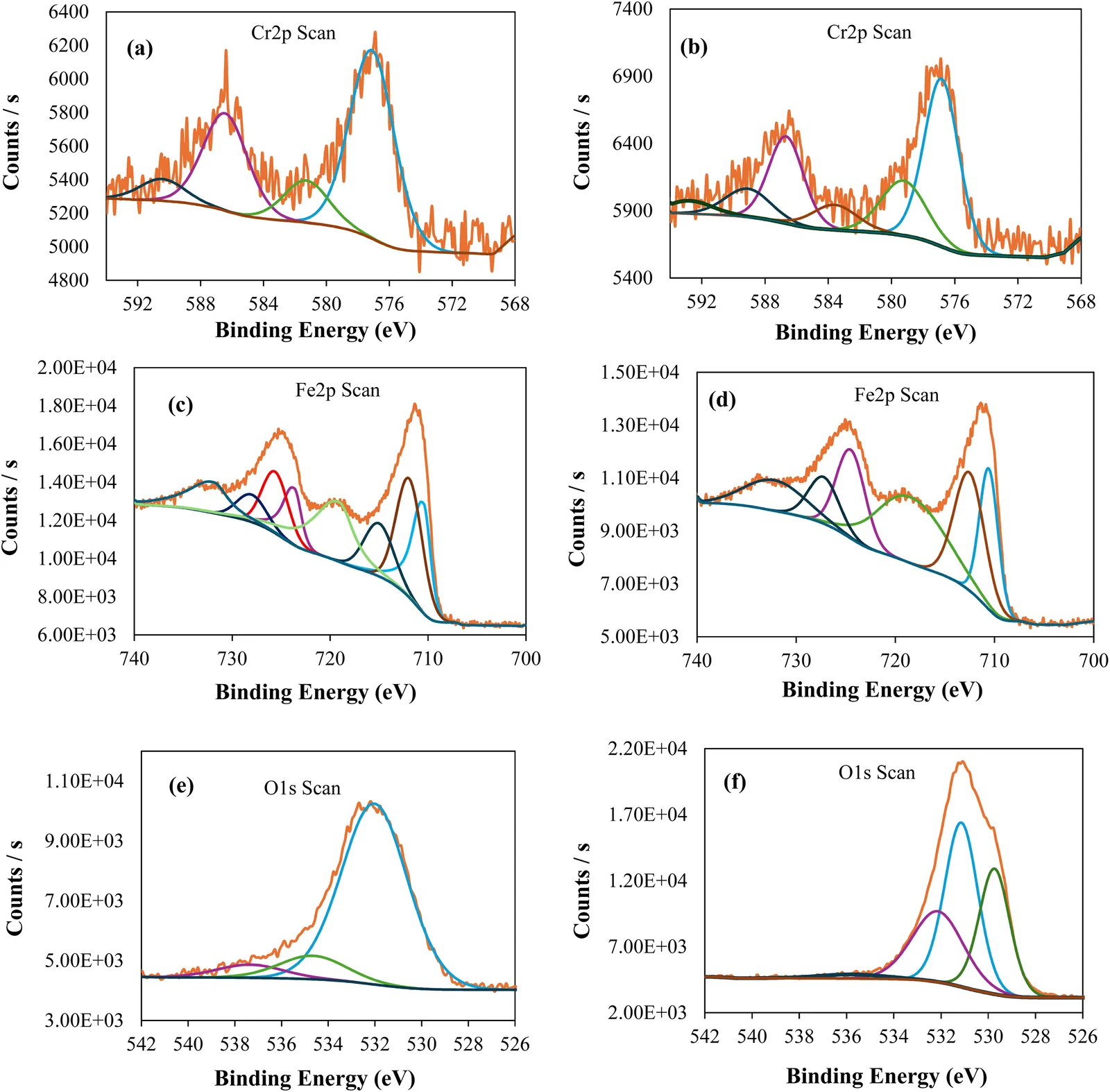
High-resolution XPS spectra of Cr 2p for (a) CB-AC and (b) nZVI@CB-AC after adsorption; Fe 2p for nZVI@CB-AC (c) before and (d) after adsorption; and O 1s for (e) CB-AC and (f) nZVI@CB-AC after adsorption.

Fig. 3 presents the XPS spectra of CB-AC and nZVI@CB-AC. According to survey spectra, C 1s (∼285 eV) and O 1s (∼531 eV) signals were detected in all samples, whereas nZVI@CB-AC additionally exhibited Fe 2p peaks (∼710–725 eV), confirming the successful incorporation of iron species (Fig. S2). No Cr signal was observed before adsorption, while distinct Cr 2p peaks appeared after adsorption, confirming chromium immobilization on both adsorbents.

The high-resolution C 1s spectrum of both adsorbents (Fig. S1) showed a peak at approximately 284.3 eV. This peak was attributed to sp^2^-hybridized C=C bonds, indicating the aromatic nature of the aromatic carbon structure. In addition, deconvolution peaks observed at higher binding energies were assigned to O–C=O (∼288.7 eV) and a high-binding-energy component around ∼292–293 eV, which is commonly associated with π–π* shake-up satellites, indicating conjugated/aromatic structures. These features confirm the presence of oxygen-containing functional groups on the carbon surface.^34^ After adsorption, the C–C/C=C component remained dominant, while slight changes in the oxygen-containing carbon species suggested their involvement in chromium binding. The O 1s spectrum of fresh CB-AC (Fig. S2a) sample exhibited peaks at 531.8 (ref. 37–39) and 533.2 eV, corresponding to hydroxyl-related oxygen species and C–O groups, respectively, which also indicates the presence of oxygen-containing surface functionalities.

For nZVI@CB-AC (Fig. S2b), three components at ∼530.0 eV, 531.2 eV, and 533 eV were assigned to oxygen in M–O bonds, hydroxyl (O–H) groups and/or M–O–C bonds, and C–O bonds, respectively. The presence of the M–O peaks confirmed that nZVI particles were successfully loaded onto CB-AC, with changes in surface chemistry, including the formation of Fe–O–C bonds.^22^ This is also confirmed by the Fe 2p spectrum of fresh nZVI@CB-AC (Fig. 3c).^35^ The Fe 2p_3/2_ and Fe 2p_1/2_ peaks at 711.0 eV and 725.0 eV show the presence of oxidized iron. The main iron form was Fe^3+^.^36^ The Fe^0^ peak, usually observed around 706.5 eV, is either very weak or absent, likely because the zero-valent iron was partially oxidized during air exposure before the XPS measurement.^15^ A small peak around 714.8 eV was derived from Fe^2+^, while the deconvolution peak at around 719.5 eV is linked to Fe^3+^. This phenomenon indicated both Fe^2+^ and Fe^3+^ were present, which was typical for magnetite-like (Fe_3_O_4_) materials and this observation is also supported by previously reported studies in the literature.^36^

Following Cr(vi) adsorption of both CB-AC and nZVI@CB-AC, the dominant peaks with binding energies of Cr 2p_3/2_ (577.2 eV) and Cr 2p_1/2_ (586.6 eV) were observed in the Cr 2p spectrum (Fig. 3a and b) indicate that the adsorbed chromium is largely in the Cr(iii) form (Cr_2_O_3_/Cr(OH)_3_); however, considering that the chromium species in the initial solution is Cr(vi), this finding suggests that a significant surface reduction reaction occurred during the adsorption process. In case of CB-AC after adsorption, the presence of a prominent component attributed to lattice oxide (M–O) peak^40^ observed at ∼531.5 eV in the O 1s spectrum (Fig. 3e), indicates the formation of Cr–O bonds on the surface. When these findings are considered together, a reduction induced adsorption mechanism is proposed in which oxygen-containing functional groups (–OH, –COOH, *etc.*) on the CB-AC surface act as electron donors, reducing Cr(vi) to Cr(iii), and the resulting Cr(iii) species are immobilized on the surface through complexation/precipitation.

In case of nZVI@CB-AC, a notable change was observed in the Fe 2p spectrum (Fig. 3d), the Fe^2+^ related component at 715.2 eV (2p_3/2_) and its corresponding 2p_1/2_ component at 725.8 eV, both present before adsorption, were no longer detected after Cr(vi) adsorption. This disappearance may indicate partial oxidation of surface Fe^2+^ species to Fe^3+^ during the reaction, consistent with a mechanism in which Fe^2+^ contributes to the reduction of Cr(vi) to Cr(iii). Compared with the O 1s spectrum of fresh nZVI@CB-AC, the three components shifted to 529.8 eV, 531.2 eV, and 532.2 eV, respectively (Fig. 3f). This may suggest that surface hydroxyl groups associated with iron species participated in Cr(vi) removal *via* adsorption-reduction process. Similar to CB-AC, the Cr 2p spectrum detected on nZVI@CB-AC surface after adsorption (Fig. 3b) revealed that chromium exists not in a single oxidation state, but in two different valence states. Components were observed at 576.9 eV, 579.4 eV, and 583.7 eV in the 2p_3/2_ region, and at 586.7 eV, 589.2 eV, and 592.9 eV in the 2p_1/2_ region. The 576.9/586.7 eV pair was attributed to Cr(iii) species (*e.g.*, Cr_2_O_3_, Cr(OH)_3_), the 579.4/589.2 eV pair to Cr(vi) species (chromate/dichromate).^41–43^

Overall, the XPS results demonstrated that Cr(vi) removal by both CB-AC and nZVI@CB-AC proceeded through adsorption-reduction mechanism, as the coexistence of Cr(vi) and Cr(iii) species was observed on both adsorbent surfaces after adsorption. However, the incorporation of nZVI did not result in a higher Cr(iii) fraction or adsorption capacity than CB-AC. Instead, oxygen-containing surface functional groups on the adsorbent also played an important role in the reduction of Cr(vi). The limited contribution of Fe species may be attributed to the rapid surface oxidation of nZVI, as evidenced by the predominance of Fe^2+^/Fe^3+^ species before adsorption and the further oxidation of surface iron after adsorption.

**Fig. 4:**
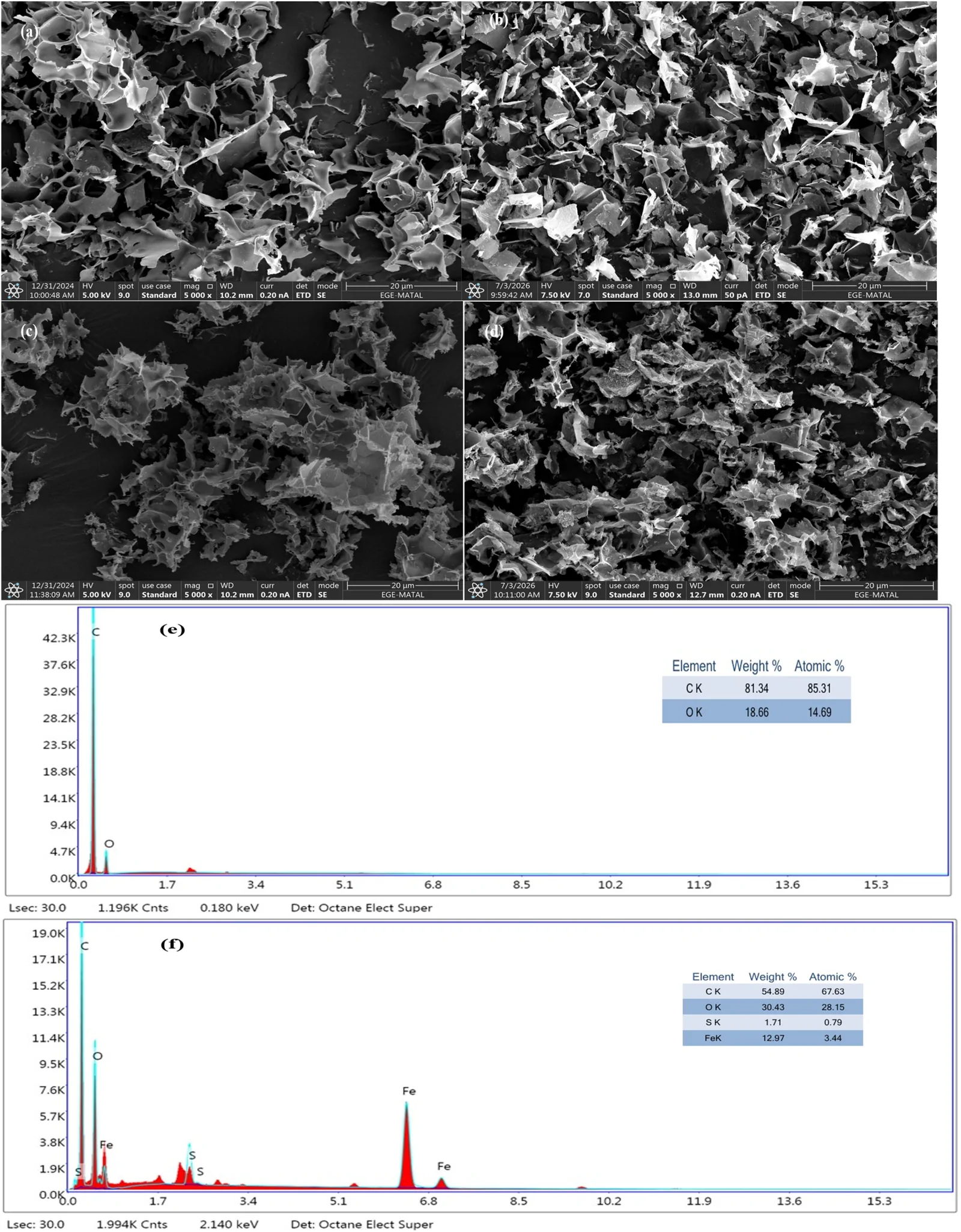
SEM images before adsorption of CB-AC (a) and nZVI@CB-AC (c); after adsorption of CB-AC (b) and nZVI@CB-AC (d) and EDX pattern before adsorption of CB-AC (e) and nZVI@CB-AC (f).

SEM images and EDX spectra of CB-AC and nZVI@CB-AC are shown in Fig. 4. In SEM images of the CB-AC (Fig. 4a), a homogeneous, highly porous structure with a flake-like morphology was observed after chemical activation with KOH. For nZVI@CB-AC (Fig. 4c), rod-like structures were visible on the surface, indicating the deposition of iron nanoparticles onto the activated carbon.^41^ The appearance of Fe peaks in the EDX spectrum (Fig. 4f) confirms that the iron modification on the activated carbon was successfully achieved. The SEM images obtained after adsorption revealed noticeable morphological changes on the adsorbent surface, indicating that the adsorption process altered the surface structure. However, no characteristic chromium peaks were detected in the EDX spectra. This is likely because the amount of chromium adsorbed under the optimum initial Cr(vi) concentration used in this study (50 mg L^−1^) was below the detection limit of the EDX technique. In addition, the homogeneous distribution and low surface concentration of chromium may have further hindered its detection by EDX. In contrast, the more surface-sensitive XPS analysis clearly confirmed the presence of chromium species on the adsorbent surface.

### 3.2 Effect of initial solution pH on removal of Cr(vi)

**Fig. 5:**
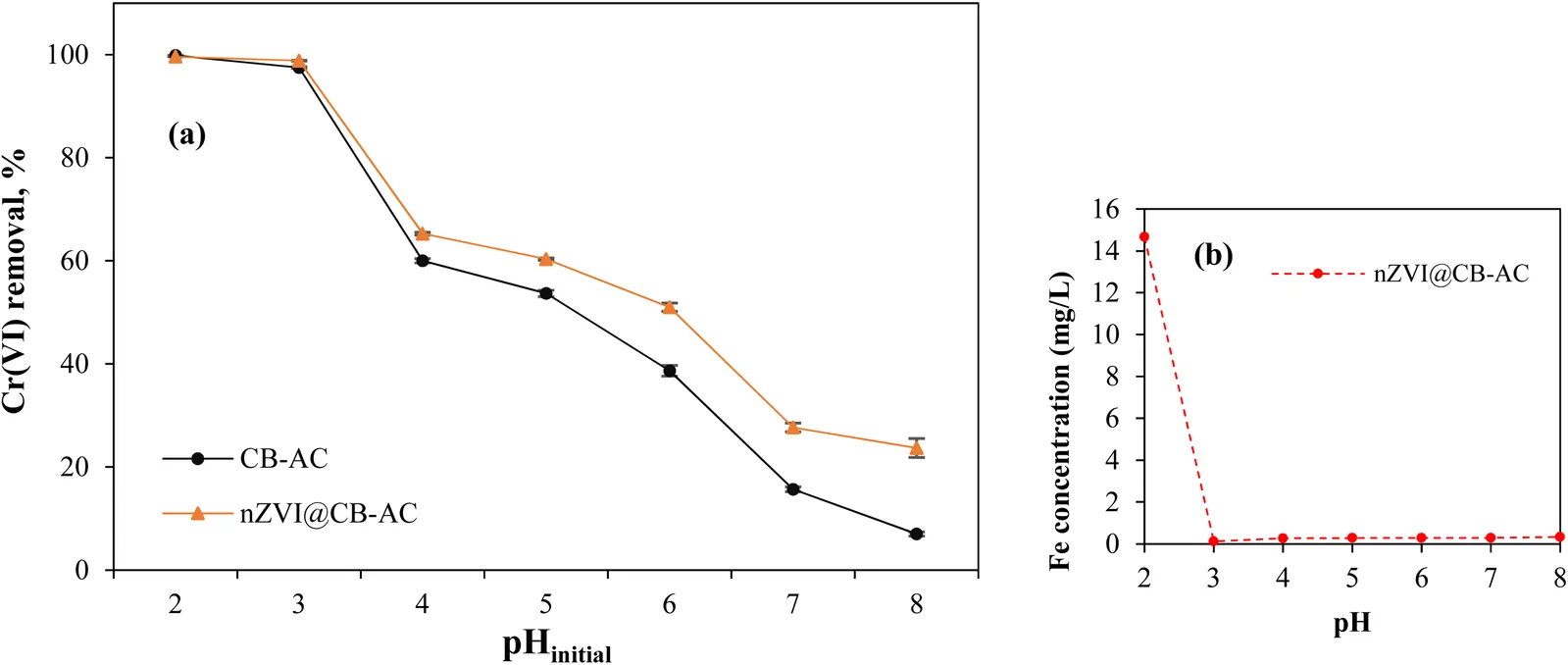
Effect of initial solution pH on (a) Cr(vi) removal efficiency of CB-AC and nZVI@CB-AC and (b) Fe concentration in solution for nZVI@CB-AC (initial Cr(vi) concentration: 50 mg L^−1^; adsorbent dosage: 1.2 g L^−1^; contact time: 24 h; temperature: 25 °C; shaking speed: 300 rpm).

The effect of initial solution pH on total Cr(vi) removal efficiency was investigated in the range of 2–8. The experiments were conducted with a 50 mg L^−1^ of initial Cr(vi) solution while keeping other parameters constant. The pH was adjusted using diluted HCl or NaOH. The relationship between total Cr(vi) removal and the initial solution pH is presented in Fig. 5.

As shown in Fig. 5, for all studied pHs, nZVI@CB-AC exhibited higher removal efficiency compared to CB-AC. Regardless of the adsorbent type, the removal of total Cr(vi) from aqueous solution was highly dependent on the pH, and the maximum adsorption occurred at pH 2 and 3. This phenomenon can be explained as follows: Cr(vi) predominantly forms neutral and anionic species, particularly chromate (CrO_4_^2−^), hydrogen chromate (HCrO_4_^−^), and dichromate (Cr_2_O_7_^2−^). Under acidic conditions (below pH 3) and at high chromium concentrations, dichromate (Cr_2_O_7_^2−^) is the dominant species, while at pH values above 6.5, Cr(vi) mainly exists in the form of chromate (CrO_4_^2−^).^44^ The anionic chromium species are favorably attracted to the protonated, positively charged surface functional groups on the adsorbent. In addition, the decrease in removal efficiency at higher pH values can be attributed to the competitive adsorption between chromate and hydroxyl ions.^34^

The leached iron concentrations after adsorption are shown in Fig. 5. Remarkably, the leached Fe concentration was 14.66 mg L^−1^ at an initial pH of 2, whereas it was only 0.12 mg L^−1^ at pH 3. This could be the result of the gradual reduction of HCrO_4_^−^ to Cr(iii) first by nZVI@CB-AC, and then, the formed Fe(ii) ions could further reduce HCrO_4_^−^ to Cr(iii).^45^

**Fig. 6:**
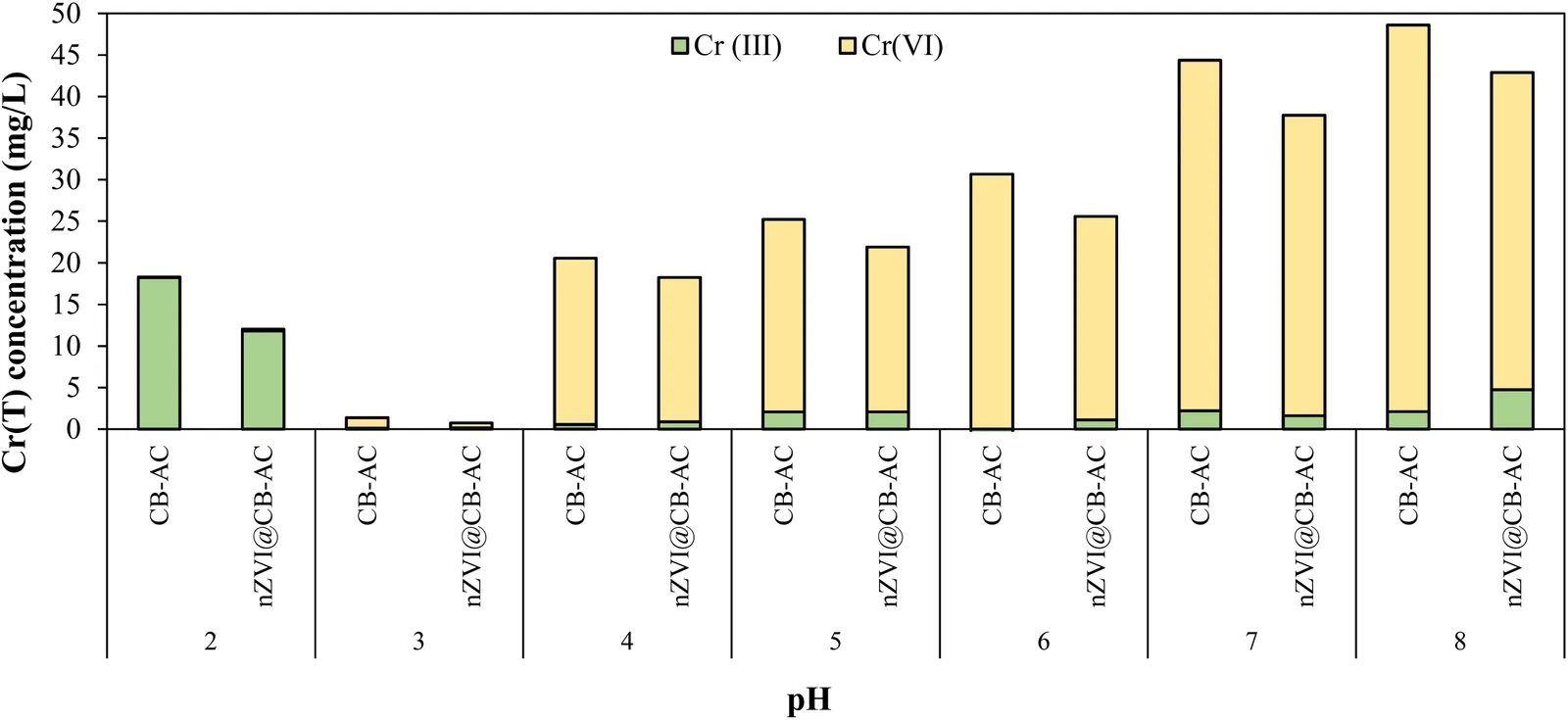
Effect of initial pH on total Cr [Cr(iii) + Cr(vi)] removal efficiency by CB-AC and nZVI@CB-AC.

The relationship between initial pH and the removal efficiency of chromium species is depicted in Fig. 6. The Cr(vi) ions were partially reduced to Cr(iii) in both adsorbents at pH 2. However, at pH 3, no Cr(iii) ions were detected in the aqueous phase. This suggested that the removal of Cr(vi) occurred primarily through two mechanisms at pH of 3: (i) adsorption of Cr(vi) species onto the active sites of the adsorbent surface, and (ii) surface-mediated reduction of Cr(vi) to Cr(iii). Considering the removal efficiency of total Cr species from aqueous phase, the optimum initial solution pH was determined to be 3.0 for both adsorbent.^45^

A similar pH-dependent trend has been reported in previous studies on nZVI-supported biochar systems. These findings indicate that Cr(vi) removal under acidic conditions is governed by a multi-step process involving electrostatic interactions and Fe^0^/Fe(ii)-mediated reduction acting synergistically. In agreement with this interpretation, Qiu *et al.* and Yi *et al.* reported that nZVI-supported biochars under low pH conditions significantly promote both Cr(vi) reduction and subsequent immobilization of the generated Cr(iii) on the adsorbent surface. Accordingly, the identification of pH 3 as the optimum condition in the present study is highly consistent with the dominant removal pathways reported in the literature.^17,46^

### 3.3 Effect of adsorbent dosage on removal of Cr(vi)

**Fig. 7:**
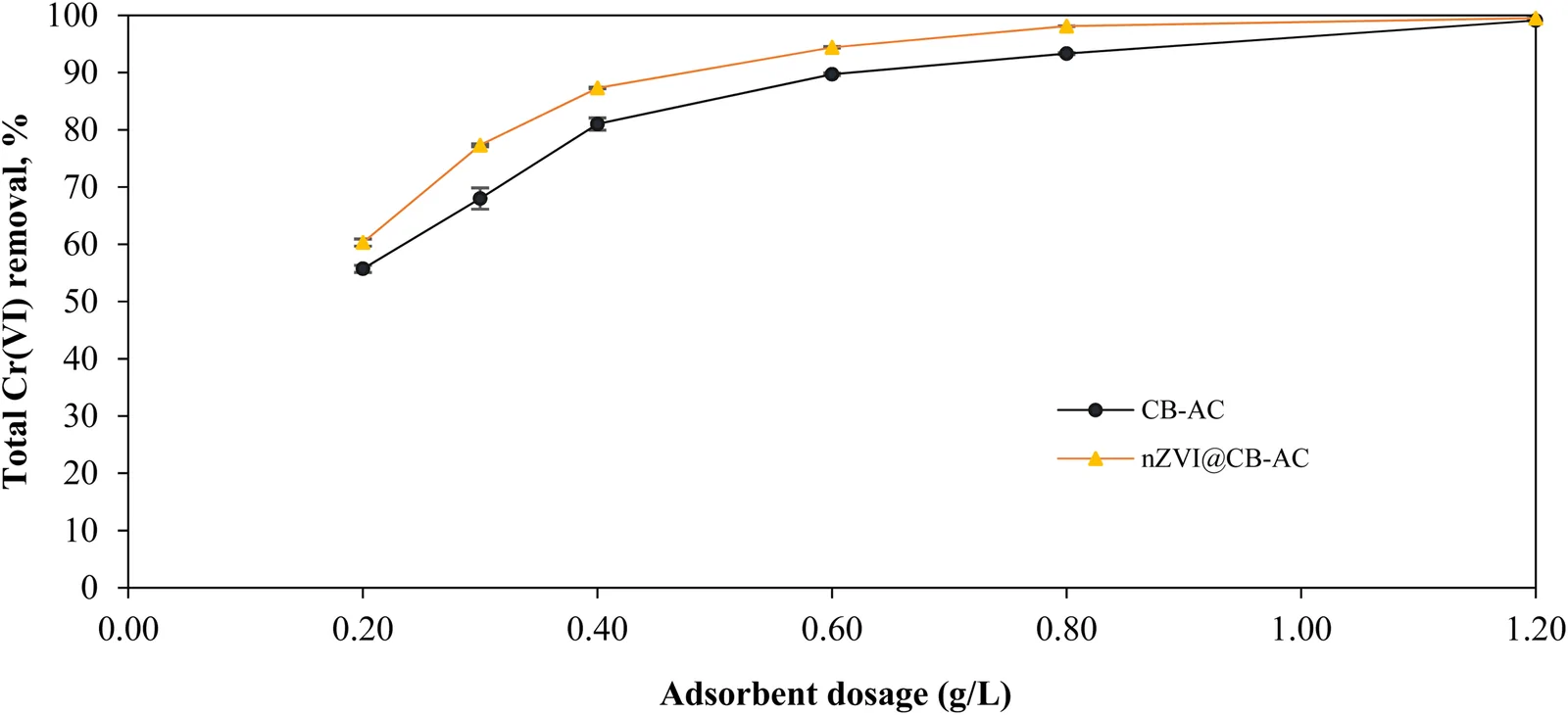
Effect of adsorbent dosage on Cr(vi) removal efficiency by CB-AC and nZVI@CB-AC. (pH: 3, initial Cr(vi) concentration 50 mg L^−1^, solution volume 25 mL, contact time: 24 h, temperature: 25 °C, shaking speed: 300 rpm).

The effect of different adsorbent dosages on the removal of total Cr(vi) ions from aqueous solutions was investigated. For this purpose, 25 mL Cr(vi) solutions with an initial concentration of 50 mg L^−1^ and adjusted to pH 3 were treated with varying amounts of CB-AC and nZVI@CB-AC adsorbents (0.005, 0.0075, 0.01, 0.015, 0.020, and 0.030 g corresponding to 0.2, 0.3, 0.4, 0.6, 0.8, and 1.2 g L^−1^, respectively). The results were presented in Fig. 7. Even at the lowest adsorbent dose of 0.2 g L^−1^, removal efficiencies of 55.7 ± 0.62% for CB-AC and 60.3 ± 0.62% for nZVI@CB-AC were achieved. With the increase in adsorbent dosage, the removal efficiency also increased, and from 0.8 g L^−1^, onwards, values above 90% were maintained for both adsorbents. This was attributed to increased available binding sites of the adsorbent by increased adsorbent amounts.^47^ Considering the high toxicity of Cr(vi) ions, an adsorbent dosage of 1.2 g L^−1^, which provided over 99% removal efficiency, was selected as the optimum condition. In terms of the adsorbent dosage–removal relationship, approximately 63% Cr(vi) removal was reported for longan seed activated carbon (LSAC) at an adsorbent dosage of about 6 g L^−1^,^48^ whereas in this study, CB-AC and nZVI@CB-AC achieved over 90% removal efficiencies at a much lower dosage of around 0.6 g L^−1^.

### 3.4 Effect of contact time on removal of Cr(vi) and adsorption kinetics

**Fig. 8:**
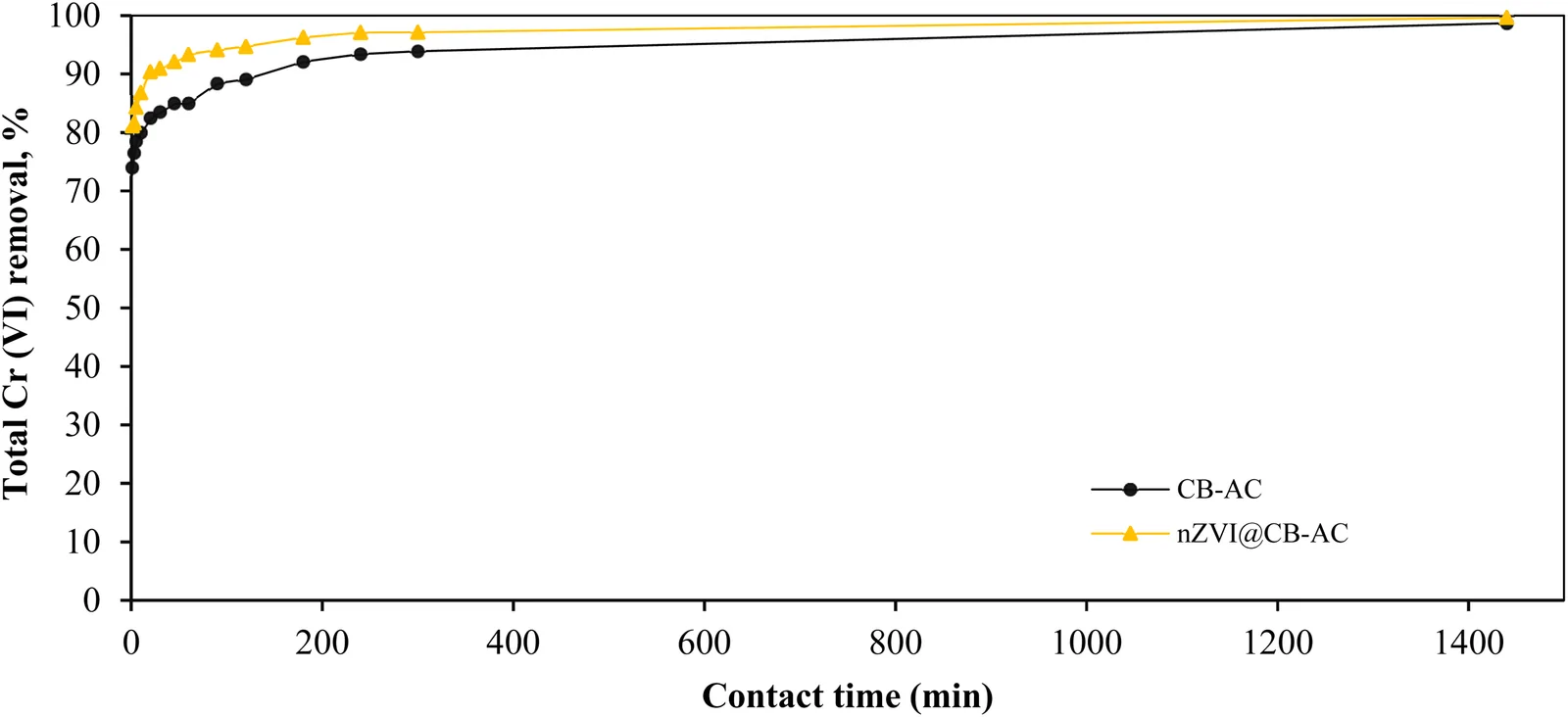
Effect of contact time on Cr(vi) removal efficiency by CB-AC and nZVI@BC-AC. (pH: 3, initial Cr(vi) concentration 50 mg L^−1^, solution volume 25 mL, adsorbent dosage: 1.2 g L^−1^, temperature 25 °C, shaking speed: 300 rpm).

Kinetic experiments were conducted to investigate the effect of contact time on Cr(vi) removal using 1.2 g L^−1^ of each adsorbent and 25 mL of a 50 mg L^−1^ Cr(vi) solution. The residual Cr(vi) concentration was analyzed after predetermined time intervals (1, 3, 5, 10, 20, 30, 45, 60, 90, 120, 180, 240, 300, and 1440 min)) to calculate the removal efficiency. The removal efficiency as a function of contact time is presented in Fig. 8, which compares the performance of CB-AC and nZVI@CB-AC.

**Fig. 9:**
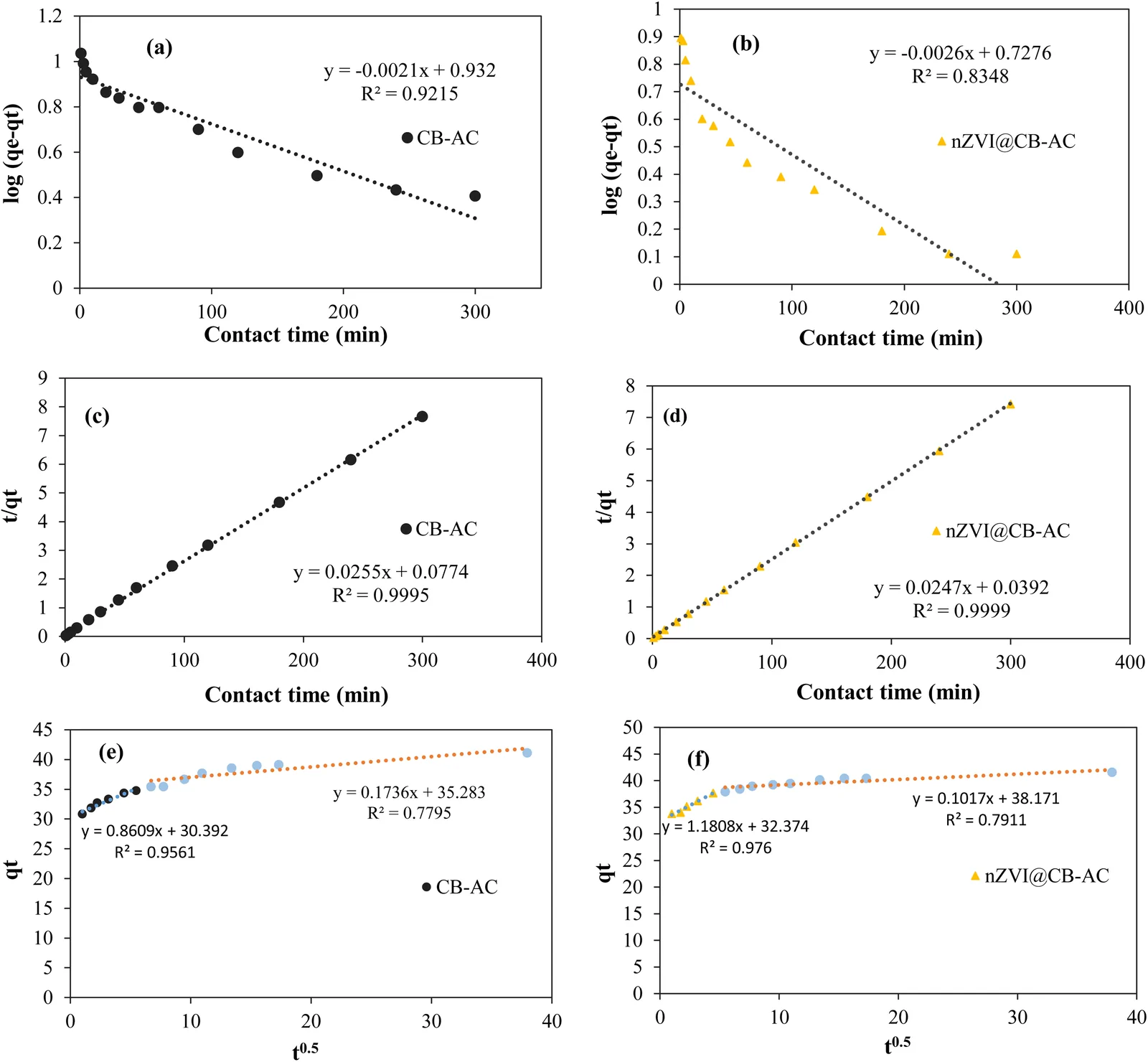
PFO plots (a and b), PSO plots (c and d), and intraparticle diffusion plots (e and f) for Cr(vi) adsorption by CB-AC and nZVI@CB-AC.

The resulting data were then fitted to the pseudo-first-order (PFO), pseudo-second-order (PSO), and intraparticle diffusion kinetic models to figure out the adsorption mechanism. The corresponding graphs are presented in Fig. 9, and the calculated kinetic parameters are presented in Table 2.

**Table 2:** Calculated kinetic parameters for Cr(vi) removal by CB-AC and nZVI@CB-AC

		CB-AC	nZVI@CB-AC	Equations
PFO	*k*_1_ (min^−1^)	0.0048	0.0060	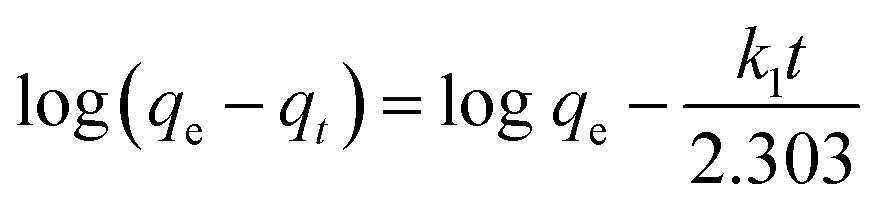
	*q*_e_cal__ (mg g^−1^)	8.5	5.3	
	*R* ^2^	0.921	0.835	
PSO	*k*_2_ (min^−1^)	0.0084	0.0156	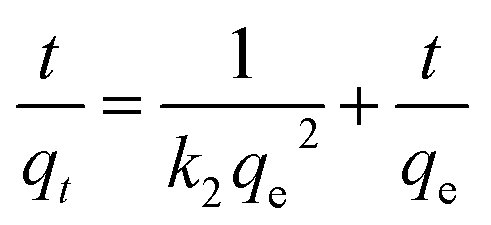
	*q*_e_cal__ (mg g^−1^)	39.2	40.5	
	*R* ^2^	0.999	0.999	
Intraparticle diffusion	*k*_id1_ (mg g^−1^ min^−0.5^)	0.861	1.182	*q*_*t*_ = *k*_id_*t*^0.5^ + *I*
	*I*_1_ (mg g^−1^)	30.392	32.374	
	*R* ^2^	0.956	0.976	
	*k*_id2_ (mg g^−1^ min^−0.5^)	0.174	0.102	
	*I*_2_ (mg g^−1^)	35.283	38.171	
	*R* ^2^	0.779	0.791	

The results revealed that nZVI@CB-AC exhibited significant advantages over CB-AC in total Cr(vi) removal. Notably, nZVI@CB-AC demonstrated faster adsorption kinetics and higher removal efficiency, especially during the initial stages. Although both adsorbents eventually achieved high efficiencies, nZVI@CB-AC maintained a superior removal rate throughout the adsorption process. This enhanced performance suggested that the incorporation of nZVI provides additional active sites and creates a synergistic effect through its reduction capabilities.^16^

Furthermore, the kinetic data for both adsorbents were better described by the PSO model, as indicated by higher correlation coefficients (*R*^2^), getting closer values for *q*_e_exp__ (41.67 mg g^−1^) and *q*_e_cal__, implying that chemisorption is the primary mechanism governing the adsorption process.^45^Fig. 9e and f depicts the intraparticle diffusion plots. Neither plot started from the origin. It showed that the diffusion was not the sole rate-limiting step; a multistep process involving film and intraparticle diffusion contributed to adsorption. The higher intercept (*I*) values indicated a more pronounced boundary layer effect, emphasizing the role of surface resistance.^49^ In the second step of ID plots, both adsorbents showed higher I and lower *K*_id_ values, suggesting intraparticle diffusion was dominant.^50^ Based on these findings, the adsorption process was governed by both chemisorption and intraparticle diffusion.

In the literature, ZnCl_2_-modified biochar^51^ and Fe_3_O_4_–onion peel biochar nanocomposites^52^ have been reported to be used for the removal of Cr(vi). They also reported the adsorption process followed the PSO kinetic model and the processes were governed by chemical interactions. Our results were consistent with the findings reported in the literature.

The superior fit of the PSO model suggests that chemisorption is the rate-limiting mechanism, and post-adsorption XPS analysis (Section 3.1 and Fig. 3) provides direct evidence supporting this interpretation. The Cr 2p spectrum of nZVI@CB-AC revealed the coexistence of Cr(vi) and Cr(iii), confirming the partial reduction of adsorbed Cr(vi). Furthermore, the disappearance of the Fe^2+^-related components at 715.2 and 725.8 eV after adsorption indicates the oxidation of Fe^2+^ to Fe^3+^, suggesting its involvement in Cr(vi) reduction. These findings are consistent with the chemical interaction step predicted by the PSO model and provide strong chemical support for this interpretation. However, they also indicate that the overall kinetic behavior results not from a single chemisorption mechanism, but from a complex mechanism involving surface reduction, complexation, and remaining electrostatic adsorption processes acting together as it was confirmed by XPS analysis.

### 3.5 Effect of initial concentration on removal of Cr(vi) and adsorption isotherms

Adsorption isotherms are used to describe the interaction of an adsorbent with an adsorbate in solution under specific conditions and to reveal the surface properties of the adsorbent. Through these models, important information can be obtained regarding adsorption capacity, surface heterogeneity, and the mechanism by which adsorption occurs.^53^ The Dubinin–Radushkevich (D–R), Freundlich and Langmuir models, which are commonly used for such evaluations, were applied to the adsorption data. The Freundlich adsorption isotherm is based on the assumption that the adsorbent surface is heterogeneous. Unlike the Langmuir model, it is not limited to monolayer adsorption and can also be applied to multilayer adsorption.^54^ The Langmuir model assumes monolayer chemical adsorption on a homogeneous surface with a limited number of sites, without interactions between molecules.^55^ The Dubinin–Radushkevich (D–R) isotherm assumes adsorption on a heterogeneous surface with a Gaussian energy distribution. D–R is not restricted to uniform adsorption sites and, unlike the Freundlich model, allows estimation of the mean adsorption energy, enabling distinction between physisorption and chemisorption.^53^

**Fig. 10:**
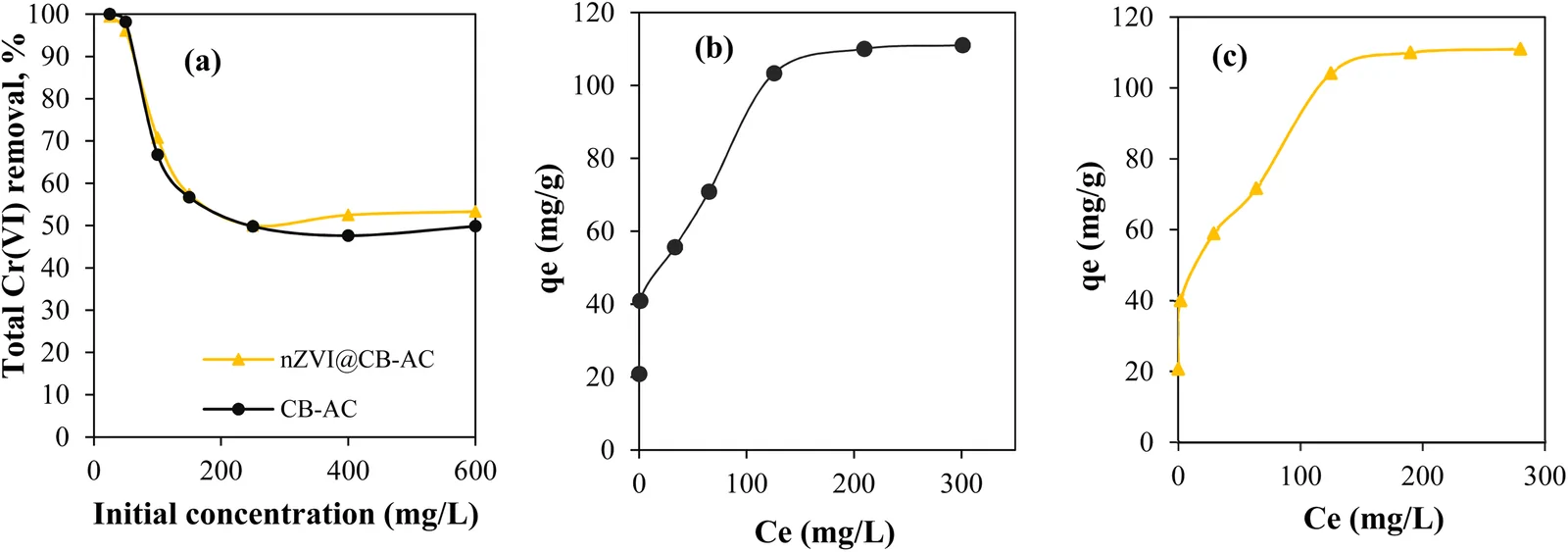
Effect of initial concentration on total Cr(vi) removal efficiency by CB-AC and nZVI@CB-AC (a) (initial concentration: 25–600 mg L^−1^, solution volume: 25 mL, pH 3, adsorbent dosage: 0.03 g, *T*: 298 K) and effect of initial concentration on adsorption capacity by CB-AC (b) and nZVI@CB-AC (c).

**Fig. 11:**
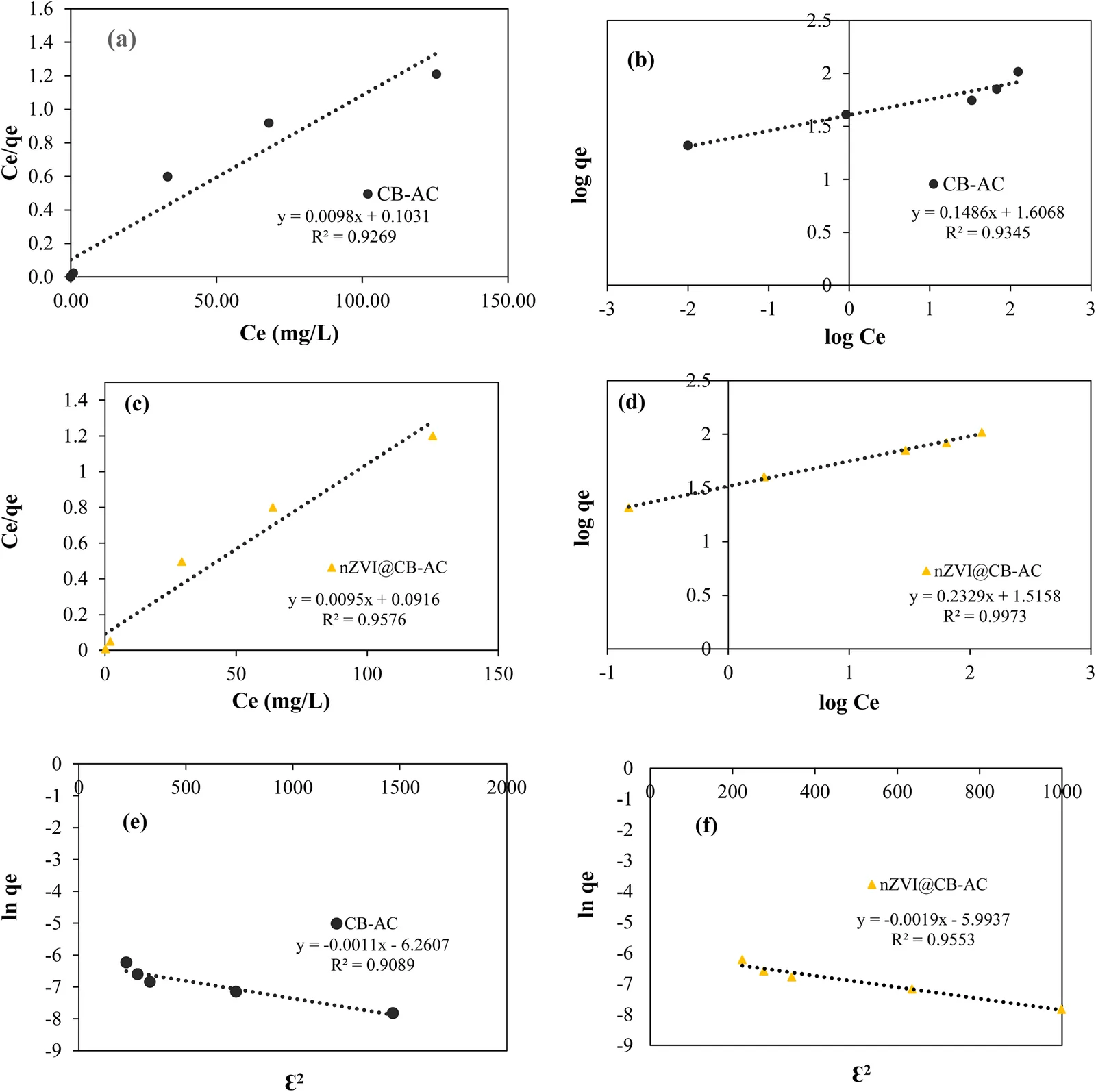
Linearized graphs of Langmuir (a and c), Freundlich (b and d) and D–R (e and f) for Cr(vi) adsorption by CB-AC and nZVI@CB-AC (pH: 3, solution volume 25 mL, C: 25–250 mg L^−1^, adsorbent dosage: 1.2 g L^−1^, temperature 25 °C, shaking speed: 300 rpm).

The adsorption capacities of CB-AC and nZVI@CB-AC for Cr(vi) were evaluated using a batch method. The effect of initial concentration on the Cr(vi) removal efficiency of the CB-AC and nZVI@CB-AC adsorbents, and the *q*_e_*versus* Ce plots are shown in Fig. 10. The equations and calculations for the isotherm models are presented in Table 3, and the related graphs are presented in Fig. 11. The Cr(vi) concentration was varied between 25 and 600 mg L^−1^, and the performance of both adsorbents was evaluated under conditions of 0.03 g adsorbent dose and pH 3.

**Table 3:** Parameters calculated by using linear isotherm models

		CB-AC	nZVI@CB-AC	Equations
Langmuir	*Q*_max_ (mg g^−1^)	101.01	105.26	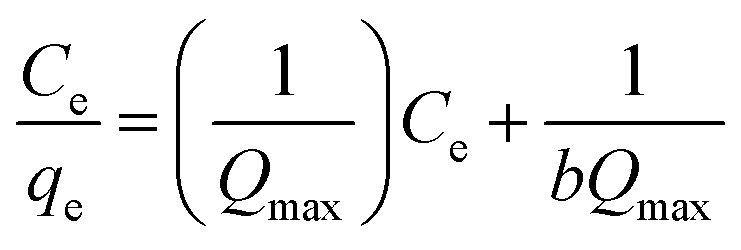
	*b* (L mg^−1^)	0.092	0.103	
	*R* ^2^	0.927	0.958	
Freundlich	1/*n*	0.15	0.233	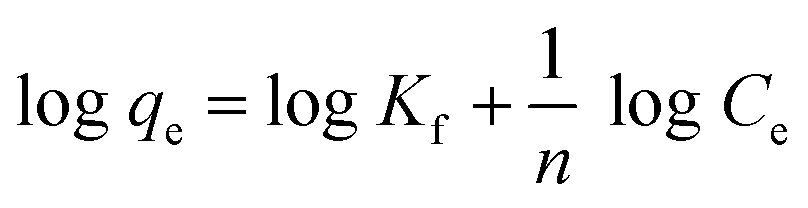
	*K* (mg g^−1^) (L mg^−1^)^1/*n*^	40.41	32.79	
	*R* ^2^	0.936	0.997	
D–R	*E* (kJ mol^−1^)	21.32	16.22	ln *Q* = ln *Q*_m_ − *kε*^2^
	*Q*_m_ (mol g^−1^)	0.0019	0.0025	
	*k* (mol^2^ kJ^−2^)	0.0011	0.0019	
	*R* ^2^	0.909	0.955	

In Fig. 10a, at low initial concentrations (25–50 mg L^−1^), both adsorbents exhibited similar high removal efficiencies. However, as the initial concentration increased, an abrupt decrease in removal efficiency was observed. At higher concentrations, the removal efficiency reached a plateau. This was due to the saturation of the vacant active sites of the adsorbents.^56^ In Fig. 10b and c, the *q*_e_*versus* Ce graphs revealed that the adsorption capacity increased with increasing initial concentration and remained constant.

In Table 3, *Q*_max_ corresponds to the maximum adsorption capacity of the CB-AC and nZVI@CB-AC (mg g^−1^) according to the Langmuir isotherm, and *b* is the Langmuir constant related to the affinity of the binding sites (L mg^−1^). For the Freundlich isotherm, *K* is the Freundlich constant related to adsorption capacity, and 1/*n* is the intensity parameter that indicates the favorability of the adsorption process. In the D–R isotherm, *Q*_m_ represents the maximum adsorption capacity (mg g^−1^), *k* is the isotherm constant related to adsorption energy (mol^2^ kJ^−2^), and *E* denotes the mean adsorption energy (kJ mol^−1^), which indicates whether the adsorption process is physical or chemical in nature.^53^

According to the parameters presented in Table 3, the experimental data for Cr(vi) adsorption fit the Freundlich isotherm model better than that of the Langmuir model. Based on the Langmuir isotherm, the maximum adsorption capacities (*Q*_max_) were calculated as 101.01 mg g^−1^ and 105.26 mg g^−1^ for CB-AC and nZVI@CB-AC, respectively. nZVI@CB-AC had slightly higher adsorption capacity owing to the modification with nZVI. Similarly, Mortazavian *et al.* reported that the modification of activated carbon *via* impregnation with nZVI, significantly enhanced the adsorption capacity compared to pristine AC (the Langmuir maximum adsorption capacity increased from 9.89 mg g^−1^ for pristine AC to 25.00 mg g^−1^ for AC/nZVI).^16^ Besides, nZVI@CB-AC exhibited a higher Langmuir constant (*b*) value than CB-AC, indicating that nZVI@CB-AC had a stronger binding affinity toward Cr(vi) ions. However, the higher *R*^2^ values obtained from the Freundlich isotherm (0.938 for CB-AC and 0.997 for nZVI@CB-AC) compared to those of the Langmuir model suggest that the assumption of a non-uniform surface with multilayer adsorption is valid for this system. Similar findings were documented in the literature.^57^ The values of *n* and 1/*n* depend on temperature and indicate adsorption conditions such as adsorption intensity or surface heterogeneity. The adsorption process is favorable if 1/*n* is greater than zero (0 < 1/*n* < 1), unfavorable if it is greater than one, and irreversible if it equals one (1/*n* = 1).^55^ Moreover, the 1/*n* values corresponding to 0.15 for CB-AC and 0.233 for nZVI@CB-AC confirmed the adsorption was favorable. These findings suggest that the incorporation of nZVI increased surface heterogeneity, allowing Cr(vi) ions to interact more effectively with the surface functional groups of the modified adsorbent.

According to the D–R isotherm model, the mean adsorption energy (*E*) values were calculated as 21.32 and 16.22 kJ mol^−1^ for CB-AC and nZVI@CB-AC, respectively. *E* values which are between 8 and 16 kJ mol^−1^, suggesting chemical sorption or ion exchange, while values below 8 kJ mol^−1^ shows physical adsorption.^53^ These energy values indicated that the adsorption process was predominantly chemisorption, involving strong interactions between Cr(vi) ions and the adsorbent surface. Overall, these results demonstrated that nZVI@CB-AC is a more effective adsorbent than CB-AC, and the adsorption of Cr(vi) ions could occur through a heterogeneous, multilayer, and chemically driven process.

The equilibrium adsorption data were also evaluated using non-linear isotherm models (Table S1). Based on higher *R*^2^ and lower RMSE/SSE values, the Freundlich model provided a better fit for both adsorbents (*R*^2^ = 0.993 and 0.997), which is consistent with the results obtained from the linearized isotherm analysis.

### 3.6 Adsorption thermodynamics

The impact of temperature on Cr(vi) removal efficiency was investigated at 25, 35, 45, and 60 °C (corresponding to 298, 308, 318, and 333 K). Thermodynamic parameters were calculated using the Van't Hoff equation, and the results are summarized in Table 4. Negative Δ*G*° values indicated that the adsorption process was spontaneous and favorable. Furthermore, the more negative Δ*G*° values with increasing temperature suggested that higher temperatures enhanced the spontaneity of adsorption. The positive Δ*H*° values revealed that the adsorption process was endothermic, while the positive Δ*S*° values indicated increased randomness at the solid–solution interface. These findings suggested that the adsorption of both CB-AC and nZVI@CB-AC increased with temperature, likely due to enhanced mobility of Cr(vi) species and greater interaction with active surface sites.^58^ Moreover, Δ*H*° values in the range of 0–8 kJ mol^−1^ are generally associated with physisorption, whereas values between 8 and 60 kJ mol^−1^ indicate a combined physico-chemical sorption mechanism. Δ*H*° values exceeding 60 kJ mol^−1^ are characteristic of chemisorption, as reported in previous studies.^19^ Also, Sun *et al.* reported that Δ*H*° values in the range of 20–400 kJ mol^−1^ are typically associated with chemisorption.^59^ In this study, the calculated Δ*H*° values for CB-AC and nZVI@CB-AC were 49.26 and 57.83 kJ mol^−1^, respectively, indicating that the Cr(vi) adsorption process is predominantly governed by a physico-chemical sorption mechanism. The thermodynamic findings contributed to a clearer understanding of the key interaction mechanisms involved in Cr(vi) removal using CB-AC and nZVI@CB-AC, and the results were found to be consistent with isotherm and kinetic model analyses. The calculated Δ*H*° values cannot be considered a definitive indicator of physical or chemical adsorption alone. When evaluated together with post-adsorption XPS analyses, it becomes clear that Cr(vi) removal is a multi-mechanism process involving electrostatic adsorption, surface reduction, and complexation acting together, rather than a single mechanism. The mixed Cr oxidation steps in XPS analysis also support this interpretation. The higher Δ*H*° value obtained for nZVI@CB-AC can be attributed to the presence of additional Fe-mediated interactions. In conclusion, thermodynamic parameters should be considered as apparent values reflecting the overall removal process, not a single adsorption mechanism. When kinetic, thermodynamic, and XPS findings are considered together, it appears that Cr(vi) removal, especially for nZVI@CB-AC, occurs *via* a multi-mechanism in which electrostatic attraction, surface reduction, and complexation act together.

**Table 4:** Calculated thermodynamic parameters for Cr(vi) removal by CB-AC and nZVI@CB-AC

			CB-AC	nZVI@CB-AC	Equations
*T* (*K*)	298	Δ*G*° (kJ mol^−1^)	−7.49	−11.81	Δ*G*° = −*RT* ln *K*_L_
	308		−8.43	−12.83	
	318		−10.17	−15.94	Δ*G*° = Δ*H*° − *T*Δ*S*°
	333		−15.14	−18.61	
		Δ*H*° (kJ mol^−1^)	49.26	57.83	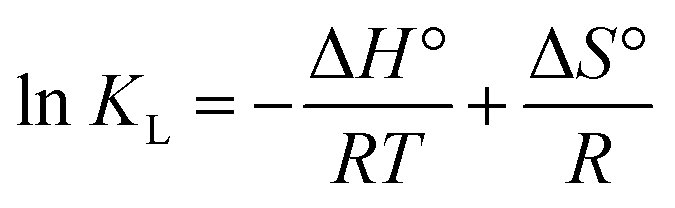
		Δ*S*° (J mol^−1^ K^−1^)	203.83	216.83	

### 3.7 Interference study

**Fig. 12:**
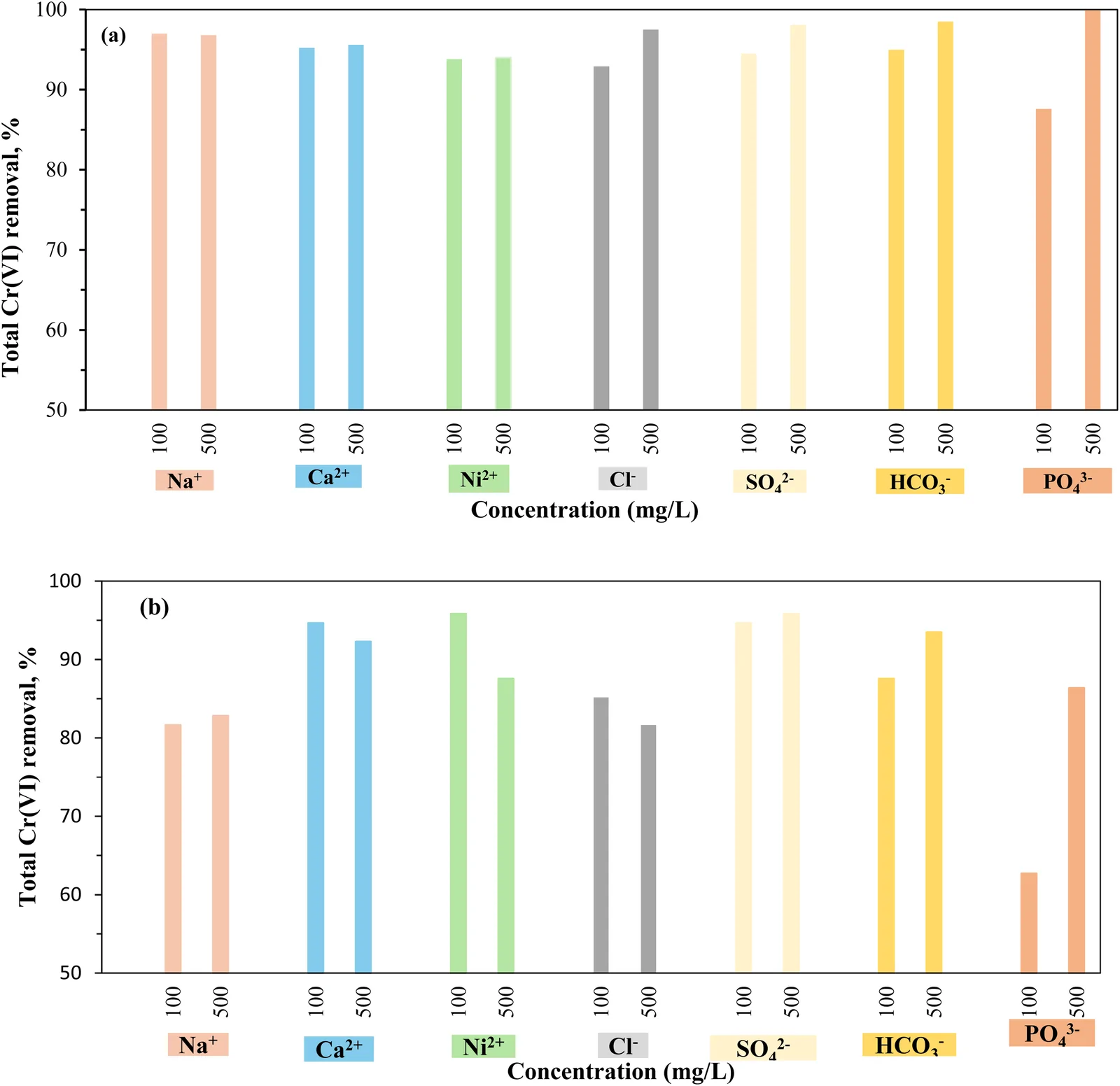
Effect of interfering ions on the removal efficiency of Cr(vi) by CB-AC (a) and nZVI@CB-AC (b).

In this study, the effects of potential interfering ions (Na^+^, Ca^2+^, Ni^2+^, Cl^−^, SO_4_^2−^, HCO_3_^−^, and PO_4_^3−^) on the removal efficiency of total Cr(vi) were investigated. Experiments were conducted for each ion at two different concentrations (100 and 500 mg L^−1^) using a 50 mg L^−1^ Cr(vi) solution under the optimum adsorption conditions. The effects of various coexisting anions and cations on total Cr(vi) removal are presented in Fig. 12. A comparison between CB-AC and nZVI@CB-AC revealed that the presence of ions affected removal efficiency to varying degrees, with a much more pronounced effect observed for the nZVI@CB-AC adsorbent. In experiments conducted with CB-AC, the effects of Na^+^, Ca^2+^, Ni^2+^, Cl^−^, SO_4_^2−^, HCO_3_^−^, and PO_4_^3−^ions were negligible. The removal efficiencies obtained above 95% for all interfering ions, except for PO_4_^3−^. The PO_4_^3−^ ion slightly reduced the removal efficiency at 100 mg L^−1^ (87.60%), whereas at 500 mg L^−1^, efficiency increased again to over 95%. This phenomenon can be explained by the competition of phosphate ions with Cr(vi) for the same active sites on the surface at low concentrations. However, at higher concentrations, PO_4_^3−^ ions may alter the electrostatic balance on the surface, facilitating Cr(vi) adsorption, or act as a pH buffer that renders the surface charge more favorable for adsorption.^60^

For the nZVI@CB-AC adsorbent, the removal efficiencies decreased considerably for Na^+^, Cl^−^, and PO_4_^3−^ ions at both studied concentrations. In the case of Ni^2+^, the removal efficiency declined to 87.54%. For Ca^2+^ and SO_4_^2−^, no considerable efficiency changes observed. The Cr(vi) removal efficiencies for low concentrations of HCO_3_^−^ and PO_4_^3−^ were suppressed more than that of the 500 mg L^−1^ of these ions. The PO_4_^3−^ ions showed the most potent inhibitory effect among all the ions tested. Due to its high negative charge (−3), phosphate exhibits a strong electrostatic attraction to positively charged Fe surfaces and may simultaneously occupy multiple active sites, thereby blocking them and significantly hindering both adsorption and electron transfer processes.^60^ The results indicated that CB-AC is more resistant to ionic interference, whereas nZVI@CB-AC is more susceptible to such interfering ions. Hence, nZVI modification enhanced reactivity but simultaneously increased sensitivity to such interferences.

### 3.8 Comparison study

The adsorption capacity of CB-AC and nZVI@CB-AC calculated by linear Langmuir equation were compared with those of various carbon-based, polymer-supported, and nZVI-containing adsorbents reported in the Table 5. It is mostly observed that activated carbons have superior performance than non-activated carbons such as biochar. CB-AC and nZVI@CB-AC (101.01 and 105.26 mg g^−1^, respectively) performed significantly better than non-activated biochar-based adsorbents (nZVI-BC (60.32 mg g^−1^), PBC (31.50 mg g^−1^), and orange peel-derived nZVI@BC (9.33 mg g^−1^)). This demonstrated that porosity caused by KOH activation is a significant factor affecting the adsorption capacity in the system. In particular, both adsorbents are produced *via* a simpler synthesis route without the need for multi-stage functionalization, exhibiting better adsorption capacity than structurally more complex engineered composites such as α-FeO(OH)/GOCS (63.19 mg g^−1^) and MCC/PANI-69 wt% (36.10 mg g^−1^). Furthermore, although PU@nZVI (600.0 mg g^−1^) has a significantly higher capacity, it relies on a synthetic polymer support instead of a low-cost waste-derived precursor, which limits its comparability in terms of circular economy. Given that cigarette butts constitute a persistent, non-biodegradable, and hazardous waste stream, converting them into a high-performance Cr(vi) adsorbent offers both environmental remediation benefits and cost advantages in terms of waste utilization.

**Table 5:** Comparison of various parameters for Cr(vi) adsorption in literature

Adsorbent	Feedstock	pH	Initial cont. (mg L^−1^)	Dose (g L^−1^)	Kinetic	Isotherm	*q*_e_ (mg g^−1^)	Reference
α-FeO(OH)/GOCS microspheres	Graphene oxide/chitosan	3.0	25	1.0	PSO	Langmuir/sips	63.19	Liu *et al.*, 2022 (ref. 61)
nZVI/N-KBC	Wheat straw	2.0	50	1.0	PSO	Langmuir	100.9	Li *et al.*, 2025 (ref. 62)
PU@nZVI	Polyurethane foam	2.0	100	0.5	PSO	Freundlich	600.0	Saad *et al.*, 2024 (ref. 63)
MCC/PANI-69 wt%	Microcrystalline cellulose	7.0	100	4.0	PSO	Langmuir	36.10	Dewa *et al.*, 2024 (ref. 64)
nZVI-BC	Sludge/sunflower seed shells	3.0	50	1.0	PSO	Langmuir	60.32	Chen *et al.*, 2022
nZVI@SBC-KOH	Sludge	5.5	50	1.0	PSO	Langmuir	63.59	Wang *et al.*, 2022
nZVI@BC (1 : 3)	Orange peel powder	2.0	20	4.0	PSO	Freundlich	9.33	Gill *et al.*, 2025
PBC	Pinecone	2.0	100	5.0	PSO	Langmuir	31.50	Masuku *et al.*, 2024
Activated magnetic biochar	Rice husk	2.0	10	1.0	PSO	Temkin	9.97	Sinha *et al.*, 2022b
nZVI@BC	Bamboo	3.0	20	0.8	PSO	Langmuir	111.99	Xu *et al.*, 2025
CB-AC	Waste cigarette butts	3	50	1.2	PSO	Freundlich	101.01	**This study**
nZVI@CB-AC	Waste cigarette butts	3	50	1.2	PSO	Freundlich	105.26	**This study**

### 3.9 Regeneration study

**Fig. 13:**
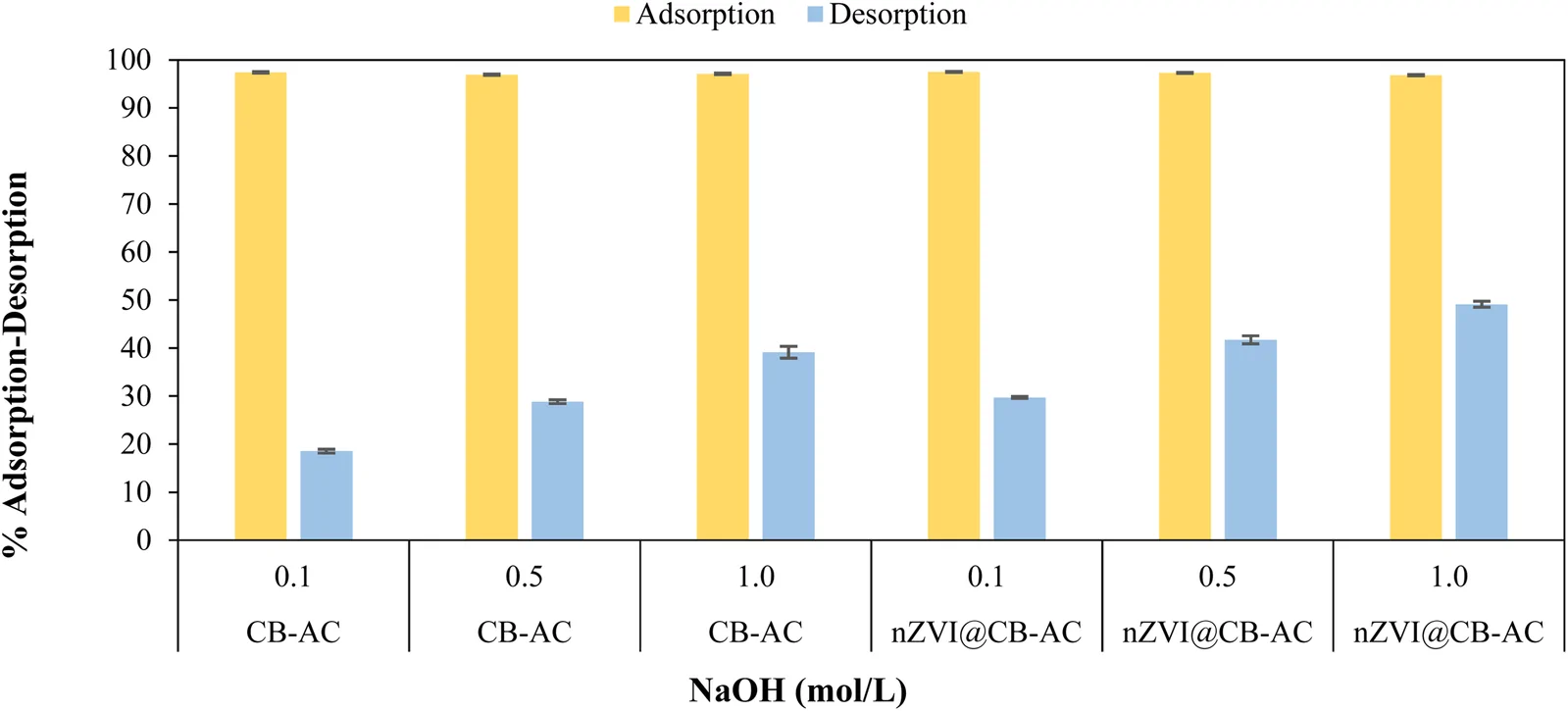
Adsorption–desorption efficiencies of CB-AC and nZVI@CB-AC for total Cr(vi) removal at various NaOH concentrations.

The recovery of Cr(vi) ions from CB-AC and nZVI@CB-AC was investigated various concentrations of HCl, H_2_SO_4_, HNO_3_, and NaOH solutions; however, no effective regeneration was achieved, and the adsorption/removal capacity of the material could not be recovered under the applied acidic conditions. Hence the study carried out by using 25 mL of NaOH solutions at concentrations of 0.1, 0.5, and 1.0 mol L^−1^. After the standard adsorption process, the adsorbents were regenerated using NaOH solutions. As discussed in the section addressing the effect of pH on removal efficiency, in aqueous media, Cr(vi) exists predominantly as HCrO_4_^−^/Cr_2_O_7_^2−^ under acidic conditions and as CrO_4_^2−^ under alkaline conditions. Since adsorption occurs mainly through electrostatic attraction between the protonated adsorbent surface at low pH and the anionic Cr(vi) species, NaOH solutions were employed in the regeneration experiments to promote surface deprotonation and to facilitate the release of CrO_4_^2−^ species back into the solution. The obtained results are presented in Fig. 13.

Even though both adsorbents regenerated with various concentrations of NaOH, after the regeneration process, the Cr(vi) adsorption efficiency remained high and stable, within the range of 93–97%. In contrast, the desorption percentages varied depending on the NaOH concentration. For CB-AC, the desorption efficiency was 18.54 ± 0.5% at 0.1 mol L^−1^ NaOH, and it reached only 39.13 ± 1.50% at 1.0 mol L^−1^ NaOH. This outcome can be attributed to the partial reduction of Cr(vi) to Cr(iii) on the activated carbon surface during adsorption, followed by precipitation or strong binding. However, a notable increase in desorption efficiency was observed for nZVI@CB-AC compared to CB-AC. The highest regeneration performance was achieved at 1.0 mol L^−1^ NaOH with a value of 49.11 ± 1.23%. This enhancement suggested that the nZVI modification altered the surface chemistry, enabling more reversible adsorption of Cr(vi) and reducing the extent of the reduction/precipitation mechanism.^49^ Overall, the results demonstrated that although Cr(vi) removal efficiency was high for both adsorbents, regeneration with NaOH was limited, while the nZVI modification significantly improved regeneration performance. Liang *et al.* reported that 0.2 M NaOH provided the highest regeneration performance for magnetic biochar, achieving a Cr(vi) desorption capacity of 8.21 mg g^−1^. After three adsorption–desorption cycles, the removal efficiency decreased from 91.12% to 80.36%, corresponding to an approximate 19.7% capacity loss.^65^ Mortazavian *et al.* investigated regeneration under deionized water, acidic, and alkaline conditions for five cycles and reported the highest Cr(vi) removal efficiency under acidic conditions. They attributed this behavior to the ability of acidic solutions to dissolve the iron oxide shell formed on the nZVI surface, thereby re-exposing reactive Fe^0^ sites and restoring the reduction reactivity toward Cr(vi).^16^ The significant decrease in removal efficiency after the second adsorption cycle suggests limited reusability of the adsorbent, likely due to partial passivation of active sites and strong binding of chromium species on the surface; therefore, a third cycle was not conducted.

### 3.10 Application studies

The performance of the CB-AC and nZVI@CB-AC adsorbents was evaluated using two different real water samples: wastewater obtained from the tanning process of the leather industry (pH 3.64) and tap water. Before testing, the water samples were filtered with blue band filter paper, and the pH was adjusted to 3 to ensure optimal conditions. To reduce the strong matrix effect, the tanning wastewater was diluted 50-fold before adsorption process. Subsequently, adsorption experiments were conducted by spiking the water samples with Cr(vi) at concentrations of 1 and 10 mg L^−1^. Considering the WHO guideline limit of 0.05 mg L^−1^ for Cr(vi) in drinking water, no higher concentrations were added.^3^

The total chromium and chromium speciation in both tap water and tanning-process wastewater were determined using ICP-MS and UV-vis spectrophotometry. The results are presented in Table 6.

**Table 6:** Cr(vi) removal performance of CB-AC and nZVI@CB-AC in real water samples

Water sample	Concentration (mg L^−1^)		Spiked concentration, Cr(vi) (mg L^−1^)	% removal, Cr(vi)	
				CB-AC	nZVI@CB-AC
Tanning process	Cr(T)	2228.19 ± 79.69	1	100	100
	Cr(vi)	—	10	100	50.00 ± 1.02
Tap	Cr(T)	—	1	100	100
	Cr(vi)	—	10	100	99.00 ± 0.12

Both adsorbents removed successfully all the added Cr(vi) at concentrations of 1 and 10 mg L^−1^. However, in the tanning-process wastewater, the removal efficiency of nZVI@CB-AC decreased to 50 ± 1.02% at the 10 mg L^−1^ spiking concentration. The high total chromium content, the presence of the matrix-derived species in the tannery wastewater, could passivate the nZVI surface, compete for active sites, or occupy the vacant sites of the adsorbent with high amounts of matrix compounds. Hence, the performance of nZVI@CB-AC was limited. In contrast, CB-AC exhibited more resilient adsorption behavior against such interferences.

## 4 Conclusions

In this study, CB-AC and nZVI@CB-AC adsorbents were successfully synthesized and comparatively evaluated for the removal of Cr(vi) ions from aqueous solutions. The results clearly demonstrate that modifying CB-AC with nZVI significantly enhanced its adsorption performance.

Characterization analyses confirmed that the presence of nZVI increased the surface area and microporosity of the adsorbent, indicating the successful formation of the nZVI@CB-AC composite. Adsorption experiments revealed that nZVI@CB-AC achieved a Cr(vi) removal efficiency of 97% at pH 3, with a contact time of 4 h and an adsorbent dose of 1.2 g L^−1^, and exhibited a high adsorption capacity of 105.26 mg g^−1^.

Kinetic studies showed that the adsorption process followed the pseudo-second-order model, suggesting chemisorption as the dominant mechanism. Isotherm analysis indicated that the Freundlich model provided the best fit, confirming the heterogeneous nature of the adsorption surface. Thermodynamic parameters further demonstrated that the adsorption process was spontaneous and endothermic, with positive entropy values indicating increased randomness at the solid–solution interface.

Overall, although CB-AC exhibited a notable ability to remove Cr(vi), the nZVI@CB-AC composite showed markedly superior adsorption efficiency, capacity, and reactivity. These findings highlight the strong potential of nZVI-modified cigarette butt–derived activated carbon as an effective and sustainable adsorbent for Cr(vi) removal from aqueous environments. Future studies should evaluate the performance of the developed adsorbent under continuous-flow conditions and in real wastewater samples to further assess its long-term stability, operational applicability, and its scale-up potential.

## Ethical approval

This article does not contain any studies with human participants or animals performed by any of the authors.

## Author contributions

Türkay Duru: investigation, formal analysis, data curation, writing – original draft. Gözde Duman: writing – review & editing, data curation. Yasemin Işlek Coşkun: writing – review & editing, methodology. Nur Aksuner: writing – review & editing, supervision, resources, conceptualization.

## Conflicts of interest

The authors have no relevant financial or non-financial interests to disclose.

## Supplementary Material

RA-016-D6RA03709G-s001

## Data Availability

This manuscript contains all relevant data produced or analyzed during this study or it contains data analyzed during this study. The data supporting this article have been included as part of the supplementary information (SI). Supplementary information is available. See DOI: https://doi.org/10.1039/d6ra03709g.
