# Supplementary material for: Cr(vi) removal performance of activated carbon (CB-AC) and nZVI-modified activated carbon (nZVI@CB-AC) derived from waste cigarette butts in aqueous solutions
Source: RSC Adv. 2026 Aug 4;16(39):44597–615. doi: 10.1039/d6ra03709g (PMC13435045; doi:10.1039/d6ra03709g)
Supplement: Supplementary file 1 [file RA-016-D6RA03709G-s001.pdf]

Supplementary Information (SI) for RSC Advances.
This journal is © The Royal Society of Chemistry 2026

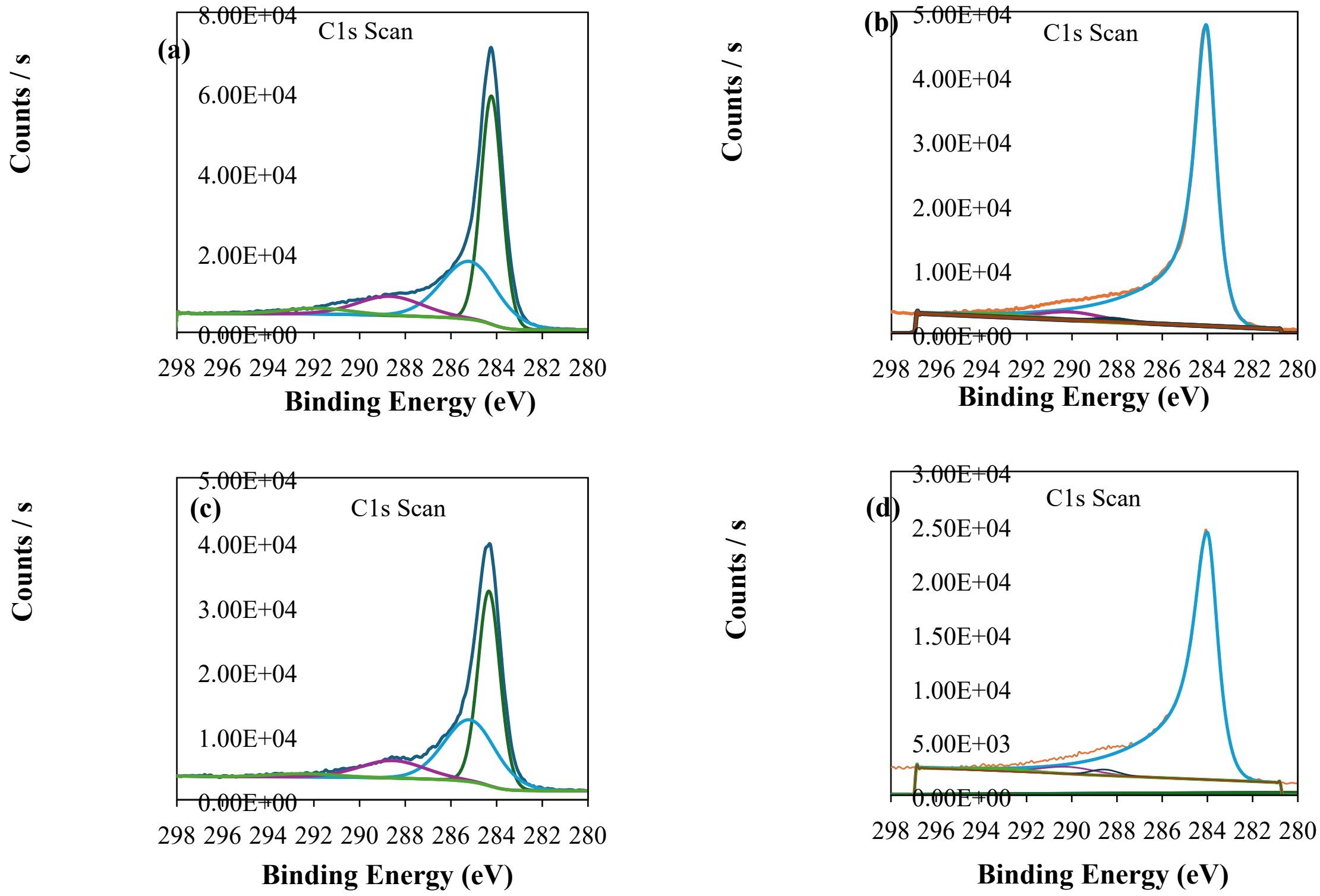

Fig. S1 High resolution XPS spectra of C1s for CB-AC (a)before and (b) after adsorption; for nZVI@CB-AC (c) before and (d) after adsorption.

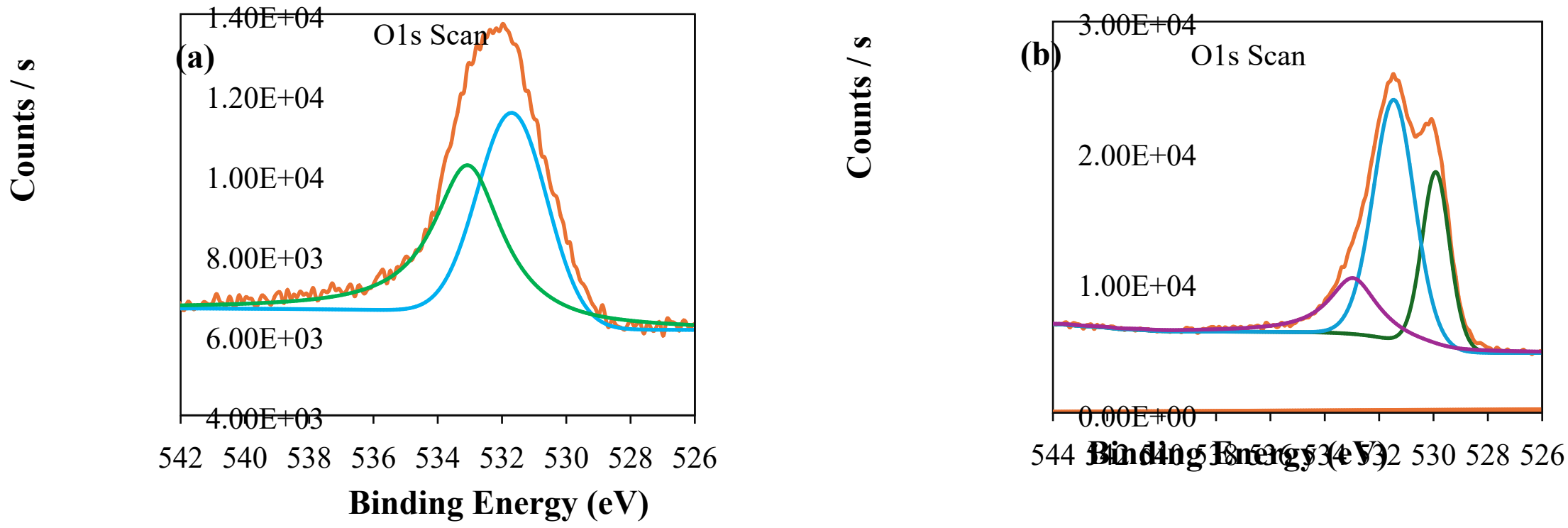

Fig. S2 High resolution XPS spectra of O1s for CB-AC (a) and nZVI@CB-AC (b) before adsorption.

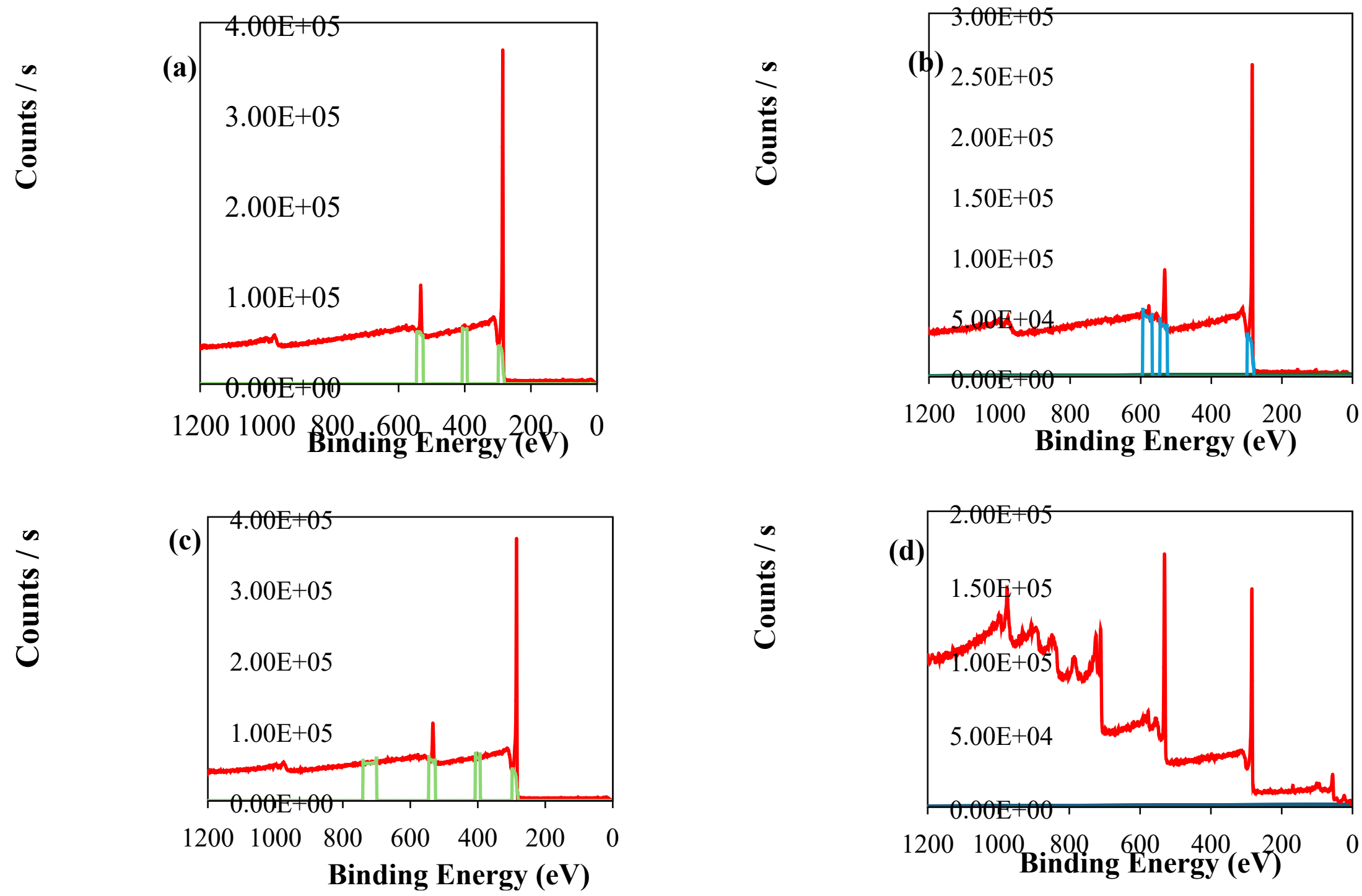

Fig. S3 XPS survey spectra of CB-AC (a) before and (b) after adsorption, and nZVI@CB-AC (c) before and (d) after adsorption.

Table S1 Isotherm parameters calculated by using non-linear isotherm models

		CB-AC	nZVI@CB-AC	Equations
Langmuir	q_{max} (mg/g)	77.37	82.07	$q_e = \frac{q_{max} \times K_L \times C_e}{1 + K_L \times C_e}$
	K_L (L/mg)	1.176	0.482	
	R^2	0.982	0.987	
	RMSE	17.77	15.18	
	SSE	1579.12	1153.24	
Freundlich	n	5.48	4.07	$q_e = K_F \times {C_e}^{1/n}$
	K_F (mg/g) (L/mg)$^{1/n}$	36.26	28.87	
	R^2	0.993	0.997	
	RMSE	10.83	7.19	
	SSE	586.49	259.00	
